# Impact of COVID-19 pandemic on elective care backlog trends, recovery efforts, and capacity needs to address backlogs in Scotland (2013–2023): a descriptive analysis and modelling study

**DOI:** 10.1016/j.lanepe.2024.101188

**Published:** 2025-01-09

**Authors:** Syed Ahmar Shah, Karen Jeffrey, Chris Robertson, Aziz Sheikh

**Affiliations:** a Usher Institute, The University of Edinburgh, Edinburgh, UK; b Department of Mathematics and Statistics, University of Strathclyde, Glasgow, UK; c Nuffield Department of Primary Care Health Sciences, University of Oxford, UK

**Keywords:** Public health, NHS Scotland, Health policy, Elective care, Healthcare systems modelling, Real-world data, Multivariable prediction models

## Abstract

**Background:**

Prioritisation of COVID-19 care led to widespread cancellations of elective care, creating a substantial backlog for healthcare systems worldwide. While the pandemic's impacts on elective hospital waiting lists during the early phase of the pandemic have been described in multiple countries, there is limited research on longer-term impacts and recovery efforts.

**Methods:**

We conducted a country-wide analysis of Scotland's healthcare system over an 11-year period (January 1, 2013–December 31, 2023) to assess the pandemic's impact on the elective care backlog, evaluate recovery efforts, and estimate the capacity increase required to clear the backlog. Our analysis involved assessments at national, elective type, regional, and specialty levels. We used descriptive statistics to compare trends and a statistical modelling approach (Vector Autoregressive model with exogenous variables) to estimate capacity increases needed.

**Findings:**

Waiting lists gradually increased before the pandemic (2013: n = 285,149; 2019: n = 385,859; 35.3% increase over six years) and then rose rapidly during the pandemic (2023: n = 667,749; 73.1% increase over four years). Capacity for elective care dropped substantially during the initial lockdown period (April–June 2020) and had not fully recovered by the end of 2023. These patterns were broadly consistent across Scotland and similar trends were observed when stratified by elective type, region, and specialty. The number of referrals waiting over a year increased from 3056 on December 31, 2019, to 78,243 (>2400% increase) by December 31, 2023. To eliminate the backlog created during the pandemic, a gradual increase in capacity, accumulating to 20% over three years is required. This corresponds to an annual increase of approximately 6.67%, translating to an additional 32,302 cases per year.

**Interpretation:**

Scotland's healthcare system struggled to meet elective care demand pre-pandemic, and the pandemic has worsened an already difficult situation. Pre-pandemic elective care capacity had not been restored by the end of 2023. While substantial additional capacity is necessary, it is crucial to adopt broader system-level strategies to effectively address waiting list backlogs.

**Funding:**

University of Edinburgh’s Chancellor Fellowship; 10.13039/501100023699Health Data Research UK.

**Research in context**

**Evidence before this study**

We searched PubMed on April 16, 2024, using the query “(COVID-19 OR Pandemic) AND (waiting OR backlog) AND elective”, with no language restrictions, limiting the search span to [Title], which identified 22 studies. After screening each study's abstract, we identified 10 relevant studies.

All studies from a range of countries (i.e. Canada, Denmark England, Ethiopia, Finland, France, Hungary, Germany, Italy, Lithuania, Poland, Spain, Sweden, Switzerland) reported a substantial impact of the COVID-19 pandemic on provision of elective care. However, most studies focused on a single medical specialty, a specific region, or only the initial lockdown phase of the pandemic.

In response to the large number of elective backlogs created during the pandemic, governments in many countries have developed and implemented recovery plans, but it is unclear to what extent these have been successful. To our knowledge, only two previous studies have developed projection models to estimate the capacity increases needed to address this backlog. Both studies, using data from the early pandemic phase (up to October 2022 in our previous study and August 2021 in the study by Howlett et al.), analysed data from NHS England. Accurately estimating future demand and the capacity increase needed to address the backlog during the pandemic's early phases proved challenging. However, with over three years having passed since the onset of the pandemic, we are now better positioned to assess the effectiveness of recovery efforts, understand how patient demand has evolved, and the capacity increase needed to address the elective care backlog.

**Added value of this study**

We analysed data from across Scotland, generating a comprehensive national picture of how the pandemic impacted waiting times for elective health services. By examining trends at the regional and specialty levels, our analysis provides much more granular insights than have been obtained from previous studies. Utilising data up to the end of 2023, our findings reveal that the NHS in Scotland had not been able to return to its pre-pandemic capacity by the end of December 2023. We observed a substantial and rapid increase in the number of referrals on the waiting list reaching 667,749 by the end of December 2023 (a 73.1% increase in four years from 2019 to 2023 compared to a 35% increase in the six years from 2013 to 2019). Between December 31, 2019, and December 31, 2023, the number of referrals awaiting resolution for over a year increased by over 2400% from 3056 to 78,282.

Our study leveraged a statistical modelling approach to estimate the capacity increase needed to clear this backlog created during the pandemic. Our modelling suggests that a sustained, gradual increase in elective capacity is needed, which accumulates to 20% in total over three years, to eliminate the backlog. This corresponds to an annual increase of approximately 6.67%, translating to an additional 32,302 cases per year.

Addressing some of the key limitations identified in previous research, our study design offers a replicable framework for other countries. By making all data publicly available, including the raw data, processed data, and the code used to generate the results in this study, we ensure transparency and promote reproducibility.

**Implications of all the available evidence**

The pandemic has added enormous pressures on NHS Scotland's attempts to manage elective hospital care in a timely fashion. Four years after the onset of the pandemic, we found insufficient progress in addressing the ongoing challenges. There needs to be substantial increases in hospital elective care capacity, sustained over several years, to clear this backlog. A holistic approach that incorporates system-level strategies, such as demand management, prioritisation of cases based on clinical urgency, and improvements in efficiency, will be essential for long-term recovery.

## Introduction

The COVID-19 pandemic led to unprecedented challenges on health systems, with many countries struggling to cope with the surge in demand resulting from COVID-19 and the extensive healthcare disruption resulting from the need to protect patients and staff.^1^^,^^2^ In Scotland, as in many other countries, this disruption resulted in postponing elective care to free up capacity for COVID-19 patients, creating a significant backlog in elective care.^3^, ^4^, ^5^, ^6^, ^7^, ^8^ Consequently, studies have reported a substantial impact on healthcare access with some groups–particularly those from lower socioeconomic backgrounds, ethnic minorities, and patients with comorbidities–disproportionately affected.^9^, ^10^, ^11^, ^12^ Emerging concerns now highlight potential long-term health consequences,^13^^,^^14^ including excess mortality rates.^15^

Addressing the backlog of elective hospital procedures is now a priority in many countries.^16^, ^17^, ^18^ In Scotland, the National Health Service (NHS) provides universal healthcare funded by general taxation, with responsibility for planning and delivering health services devolved to regional health boards. With a population of approximately 5.5 million, Scotland's health system operates across various levels of care, including primary, secondary, and tertiary services, and has faced substantial strain in maintaining healthcare capacity during the pandemic.^11^ Understanding how health systems like Scotland's NHS have performed during the pandemic is key to developing effective recovery strategies. This includes exploring the concept of health system resilience, which refers to the capacity of a health system to prepare for, manage (absorb, adapt, and transform), and recover from crises such as pandemics.^19^

To effectively tackle the elective care backlog, it is essential to understand the pandemic's impact on healthcare system performance at national, regional, and specialty level; create recovery plans; proactively monitor progress; and make data readily available to policymakers in a clear format to support data-driven decision-making. However, much of the existing research focuses primarily on the pandemic's impact on elective care without sufficiently addressing the actions needed for recovery, often limiting analysis to specific segments of the health system or certain specialties.^4^^,^^20^, ^21^, ^22^, ^23^, ^24^ This narrow scope hinders the development of effective overall recovery plans.

To address these gaps, we sought to investigate the performance of Scotland's National Health Service (NHS) throughout the pandemic. Specifically, we aimed to analyse trends in pending elective care across Scotland (nationally and stratified by elective type, region, and specialty) to assess the pandemic's impact, assess NHS's recovery progress, and estimate capacity increases needed to address the backlog using a previously developed modelling approach.^25^

## Methods

### Data source and setting

We used data from the Public Health Scotland (PHS) Stage of Treatment waiting times dataset.^26^ This dataset is derived from data provided by each of NHS Scotland's 14 territorial health boards. PHS, a national health board funded by the government and responsible for public health nationally, maintains the centralised data repository (waiting times data mart) that houses the Stage of Treatment dataset. The Stage of Treatment dataset is published quarterly and is publicly available under the UK open government license.^27^ This dataset captures information on elective care wait times for patients accessing acute specialist services, categorised as either outpatient (non-admitted) or day cases/inpatients (all admitted cases, including both day cases and overnight stays). For brevity, we will refer to all admitted cases (day cases and inpatients) as “inpatients” henceforth.

PHS works closely with NHS Boards across Scotland to ensure the data are complete, accurate, and comprehensive, with waiting times measured according to Scottish Government guidelines. To maintain high standards of data quality, PHS adheres to the UK Statistics Authority Code of Practice,^28^ producing accredited official statistics that are independently reviewed by the Office for Statistics Regulation for trustworthiness, quality, and value. Additionally, PHS publishes quarterly updates and provides a public log of data quality issues to ensure transparency.

We accessed the publicly available data from the PHS website covering 11 years (January 1, 2013–December 31, 2023). Full-year data is available starting from 2013, which is why this period was selected, and the latest data extends to the end of 2023. We analysed datasets covering overall capacity (number of referrals handled, which included patients treated or removed for various reasons, such as transferred, treatment no longer required, or death); ongoing waits (number of patients waiting for treatment); distribution of wait times by duration; and additions and removals from waiting lists (Supplementary Materials, Section 1). The definition of capacity, in this study, reflects the total number of cases the system is able to process, not just those treated, and represents the realised output (throughput) rather than potential output. While capacity is traditionally viewed as potential output, we adopt a pragmatic approach to account for all removals from the system. The specific datasets downloaded can be found on GitHub (link: https://github.com/syedahmar/ElectiveCare-Scotland).

### Study design

We employed a two-part approach. First, we conducted a descriptive analysis of country-wide aggregate data on elective care waiting times in Scotland and assessed the extent of recovery against the target set by the Scottish Government. We then developed a statistical model using these data to project the capacity increase needed to reduce the backlog to pre-pandemic level (i.e. clear the backlog created during the pandemic).

### Outcome measures

We utilised several outcome measures to assess elective waiting times and capacity. These were: total pending referrals at any given point during the study period; wait duration (the number of weeks a patient had been waiting for treatment following a referral); and total referrals resolved (a referral was considered resolved once a referred patient was first seen by a consultant/admitted).

### Data analysis

To assess trends over time, the impact of the COVID-19 pandemic, and the capacity increase needed to address the backlog, we employed several analytical methods (detailed below). We first analysed the overall data (all elective care, and all specialties from across Scotland) and then undertook separate analyses for inpatients and outpatients.

#### Pre-pandemic and pandemic comparisons

First, we compared referral activity during the pre-pandemic period (2013–2019) with the pandemic period (2020–2023). We calculated the quarterly mean values for both pending and resolved referrals, with 95% confidence intervals (CI) while accounting for any seasonality (Supplementary Materials, Section 2). We performed this for 2013, 2019, and 2023 to assess trends. Using these means, we calculated the percentage change in referrals during the pre-pandemic and pandemic periods.

#### Wait time distribution

We calculated the percentage of ongoing waits exceeding the Scottish Government's target of 12 weeks.^29^ Additionally, we determined the total number of referrals waiting for more than a year.

#### Stratified analyses

We conducted stratified analyses by health board and specialty (Supplementary Materials, Section 3 lists the 14 boards and 49 specialties).

#### Statistical model for capacity projections

Building on our previous work using NHS England data,^25^ we employed a Vector Autoregressive with exogenous variables (VARX) model to assess how changes in referral volume might impact future waiting times. This model considers the total number of pending referrals in each period as a function of the total number of pending referrals in the previous period, the number of new referrals added, and the number of referrals resolved. Our statistical model is designed to be flexible, learning the inflow and outflow dynamics of the healthcare system regardless of whether total removals or treated-only cases are used as the outflow measure. Unlike a mechanistic approach, where explicit terms are required for each individual phenomenon, our model does not need to account for every possible factor directly. Instead, it captures the overall system dynamics by adjusting its parameters based on the observed data, provided there is enough flexibility to model inflow and outflow effectively. This ensures that the model can adapt to different contexts, as long as the inflow and outflow definitions remain consistent during both model development and projection phases.^30^ We chose a model order of 4 to capture any seasonal variations present in the quarterly data. The maximum likelihood method was used to estimate the best-fitting values for each model parameter, utilising all available quarterly data from January 1, 2013, to December 31, 2023 (i.e. number of referrals added, removed, and pending). The Akaike Information Criterion (AIC) was used to select the model with the optimal balance between the number of parameters and goodness of fit (a lower AIC indicates a better fit). We explored the use of different lag structures (one and four lags, and all four lags) to determine the most suitable approach for projections. Supplementary Materials, Section 4 provides further details on the model, the associated assumptions, and the process for selecting the optimal model.

We simulated multiple scenarios where healthcare capacity was increased from 0 to 25% in increments of five percentage points. For each scenario, we ran 1000 simulations and computed the 2.5th, 50th (median), and 97.5th percentiles. The 95% prediction interval was then defined as the range between the 2.5th and 97.5th percentiles surrounding the median value (50th percentile). We focus on a three-year projection, aligning with the recovery plan's timeline, while recognising that policymakers may opt for different strategies that could extend the timeframe for clearing the backlog.

#### Assessment of progress against recovery plan

In August 2021, the Scottish Government published the NHS Recovery plan 2021–2026 to tackle the backlog.^29^ The plan outlines several key actions to address the backlog, including increasing the workforce, expanding capacity in primary, community, outpatient, and inpatient care, and introducing innovative practices to enhance service delivery. While our study focuses specifically on elective care backlogs, we track progress against the plan's targets for elective services.

Defining the pre-pandemic reference levels to be 270,000 inpatient cases/year (67,500/quarter) and 1.4 million/year (350,000/quarter) for outpatients, the plan included a substantial capacity increase in the “coming years” beyond the pre-pandemic levels. More specifically, the recovery plan's target was to increase capacity to 74,375 inpatients and 364,500 outpatients in the quarter April–June 2022. Until the last quarter of 2023 (October–December 2023), the recovery plan envisaged an increase in capacity of 7625 for inpatients and 17,071 for outpatients per quarter. We will assess how much of the proposed capacity increase was achieved and compare it with these targets to evaluate the recovery efforts over the designated timeframe.

### Role of funding source

The funders had no role in study design, data collection or analysis, decision to publish, or preparation of the manuscript.

## Results

This section presents the key findings of our analyses, divided into four parts: (1) overall numbers, the extent of disruption and projections, (2) time distribution of completed cases, (3) time distribution of ongoing cases, and (4) assessment of recovery against the NHS Recovery Plan (2021–2026). The main results for each section are highlighted upfront, with further analyses provided in the Supplementary Materials. Readers interested in more detailed breakdowns can refer to these additional sections.

### Overall numbers, extent of disruption and projections

**Fig. 1:**
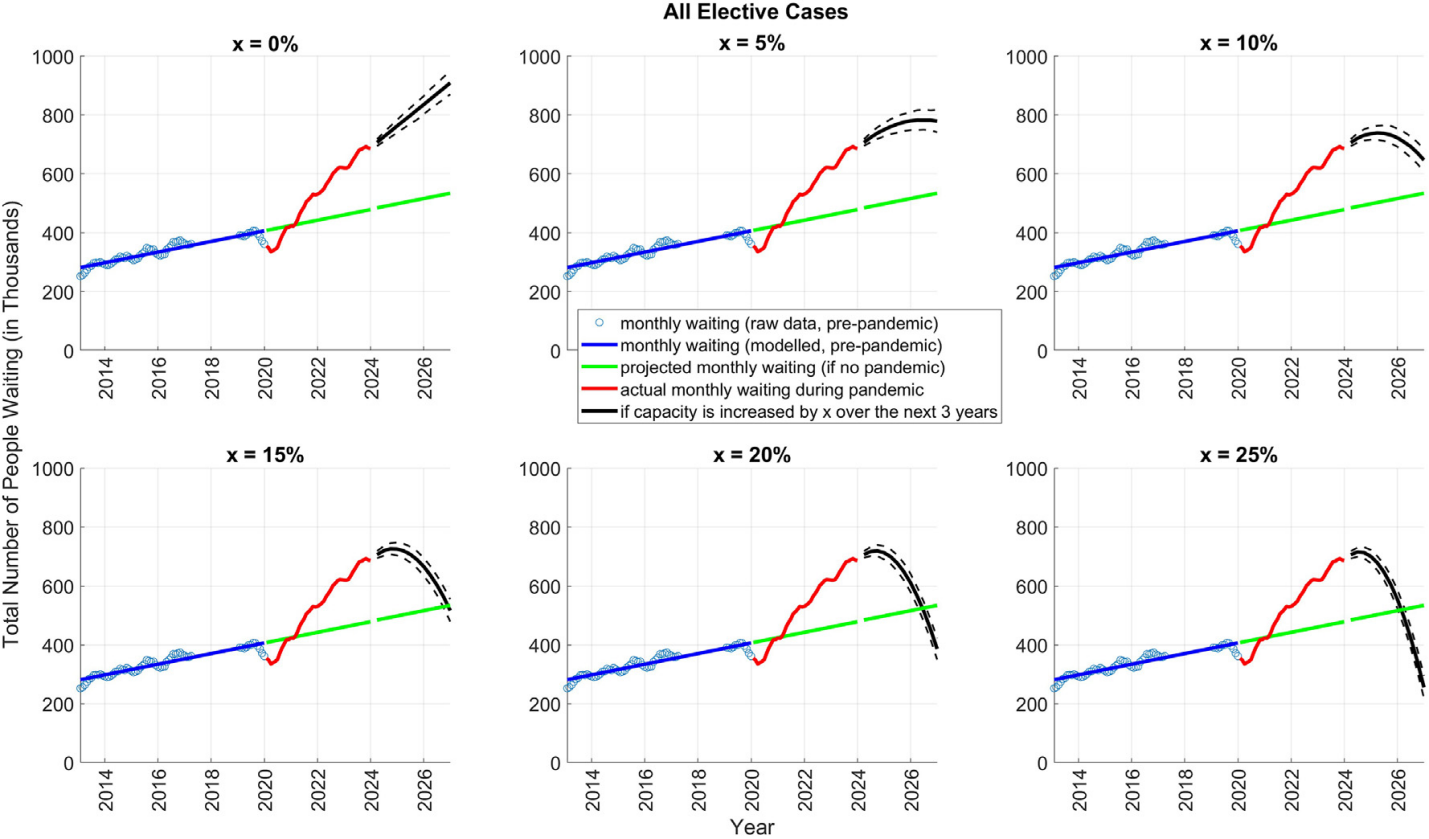
Number of Pending Referrals showing the extent of disruption and the projections. The starting capacity is based on the mean quarterly values from 2023, the most recent year with complete data.

Figure 1 shows the total number of pending appointments from January 1, 2013, to December 31, 2023, projections over the following three years (i.e. January 1, 2024–December 31, 2026) and the linear projections (the counterfactuals if the pandemic had not happened) based on the data from the pre-pandemic period (January 1, 2013, to December 31, 2019). The list grew by approximately 15,000 referrals/year in the seven years prior to the pandemic (a growth of 106,573 or a 42% increase from 254,612 on January 1, 2013, to 361,185 on December 31, 2019). In the subsequent four years, the waiting list grew by about 81,000/year (a growth of 323,166 or an 89% increase—from 361,185 on January 1, 2020, to 684,351 on December 31, 2023). Our analysis utilising the optimal VARX model (the optimal model had a lag of 1 and 4, see Supplementary Materials, Section 4 for detailed comparisons) predicts that the peak number of patients awaiting treatment will vary depending on the capacity increase implemented over the next three years. With a 5% increase, the peak is estimated at 782,526 by March 2026. A 10% increase could see the peak reach 738,959 by March 2025. Increasing capacity by 15%, 20%, or 25% could result in progressively lower peaks by September 2024 (725,512 and 718,285 respectively) or June 2024 (715,044). These projections assume a linear and gradual capacity increase starting from the mean level observed between January 1, 2023, and December 31, 2023. To fully address the backlog created during the pandemic, the capacity must increase by at least 20% in total (assuming a uniform rate of increase over time, see Figure S3, Supplementary Materials, Section 4) over the next three years compared to the mean capacity attained in 2023. A 20% increase over three years corresponds to an annual increase of approximately 6.67%, which translates to an additional 32,302 cases per year. Without any increase in capacity, total pending referrals are predicted to reach 907,853 by December 2026. In our main analysis, we assumed the demand to be equal to the mean quarterly demand in 2023. To ensure robustness, additional sensitivity analyses were conducted, where demand was assumed to vary over the 3-year projections period (−10%, −5%, +5%, and +10%). These analyses (Supplementary Material, Figure S6) indicate that to eliminate the backlog created during the pandemic, capacity needs to increase by 10%–30% depending on variations in demand. It is important to note that these estimates include error bounds, reflecting the inherent uncertainty associated with modelling real-world phenomena.

Figures S7–S8 (Supplementary Materials, Section 5) show the same analysis separately for inpatients and outpatients. The growth in the waiting list was substantially greater during the pandemic compared to the pre-pandemic period: 19,000/year versus 5000/year for inpatients; 61,000/year versus 10,000/year for outpatients. To address the pandemic-induced backlog, the total capacity must substantially increase over the period 2024–2027 compared to the mean quarterly capacity achieved during January–December 2023: 25% for inpatients; 15% for outpatients (see Supplementary Materials, Section 5 for further results).

### Time distribution of completed cases

Table 1 provides an overview of the trend in capacity and backlog comparing the pre-pandemic (2013–2019) and the pandemic period (2019–2023). While the backlog substantially increased in both periods, a concurrent decline in capacity exacerbated this trend during the pandemic.

**Table 1:** Backlog, and Capacity in 2013, 2019 and 2023 stratified by elective type (overall, outpatients and inpatients) and the corresponding percentage change from 2013 to 2019 (pre-pandemic), and from 2019 to 2023 (during pandemic) periods.

	Number of cases in thousands, mean across the four quarters in the year (95% CI)			Percentage change (%)	
	2013	2019	2023	2013–2019	2019–2023
**Overall (all elective types, all specialties and across Scotland)**					
Backlog	285.1 (256.4; 313.9)	385.9 (357.1; 414.6)	667.7 (624.2; 694.5)	35.3	73.1
Capacity	432.6 (411.9; 453.4)	437.6 (416.9; 458.4)	370.7 (345.6; 396.4)	1.2	−15.3
**Outpatients (all specialties and across Scotland)**					
Backlog	232.2 (204.8; 259.1)	308.3 (281.1; 335.4)	517.6 (476.7; 543.2)	32.9	67.9
Capacity	348.5 (332.0; 365.0)	367.0 (350.5; 383.5)	311.3 (292.2; 332.6)	5.3	−15.2
**Inpatients (all specialties and across Scotland)**					
Backlog	53.2 (51.1; 55.3)	77.6 (75.5; 79.7)	150.2 (146.8; 152.0)	45.9	93.6
Capacity	84.2 (79.2; 89.1)	70.6 (65.7; 75.5)	59.3 (52.6; 64.7)	−16.1	−16.0

Figure S9 (Supplementary Materials, Section 6) provides the distribution of completed cases and the percentage that were seen within 12 weeks of referral, during the study period. There were 413,718 appointments in the first quarter of 2013 with most (391,041; 95%) seen within 12 weeks. In the quarter just before the pandemic (October–December 2019), 434,615 appointments took place. However, by this time, the proportion of patients seen within 12 weeks of referral had fallen from 95% in the first quarter of 2013 to 76%.

Since the beginning of the pandemic, there has been a substantial decline in elective care capacity. During the April–June 2020 quarter, capacity was at the lowest point during the study period with 144,663 appointments (a drop of 67% compared to October–December 2019). There has since been some recovery, but the capacity has continued to remain below the pre-pandemic period. In the last quarter of the study period (October–December 2023), there were 376,806 appointments with 61% of patients seen within 12 weeks of referral. The same overall pattern was observed when stratified by elective type (see Figures S10 and 11, Supplementary Materials, Section 6).

### Time distribution of ongoing cases

Table 2 breaks down the trends in how long referrals have been waiting. On January 1, 2013, there were 343 pending appointments with a waiting time of over a year. This grew to 3056 by December 31, 2019 (791% increase), and to 78,282 by December 31, 2023 (over 2400% increase). On December 31, 2023, almost a quarter of inpatient referrals (36,909; 23.3%) have been pending for over a year.

**Table 2:** Total number of pending referrals, number of referrals waiting for over 12 weeks, and over 52 weeks, at the end of 2013, 2019 and 2023 stratified by elective type (overall, outpatients, and inpatients).

	Number of pending referrals		
	Total waiting in thousands	Waiting >12 weeks (% of total referrals)	Waiting >1 year (% of total referrals)
**At the end of 2013 (December 31, 2013)**			
Overall	292.7	13.0 (4.4)	0.5 (0.2)
Outpatients	236.5	12.0 (5.1)	0.5 (0.2)
Inpatients	56.1	1.0 (1.8)	0.01 (0.02)
**At the end of 2019 (December 31, 2019)**			
Overall	361.2	101.3 (28.0)	3.1 (0.8)
Outpatients	281.2	75.3 (26.8)	1.8 (0.6)
Inpatients	80.0	26.0 (32.5)	1.3 (1.6)
**At the end of 2023 (December 31, 2023)**			
Overall	684.4	421.8 (61.6)	78.3 (11.4)
Outpatients	529.3	317.1 (59.9)	42.2 (8.0)
Inpatients	155.0	104.8 (67.6)	36.1 (23.3)

Figure S12 (Supplementary Materials, Section 7) presents the distribution of ongoing cases and the percentage that were waiting for over 12 weeks. On January 1, 2013, only 3% of patients (6610 of 254,612) were waiting for over 12 weeks. This grew to 28% by December 31, 2019 (101,302 of 361,185 totals). During the pandemic, the percentage of patients who waited for longer than 12 weeks increased substantially, reaching 62% on December 31, 2023 (421,839 of 684,351 totals). Figures S13–S14 shows the distribution of cases stratified by elective type (see Supplementary Section 7 for further details).

Significant differences were observed across regions and specialties in inpatient waiting times. The worst-affected health boards, with over a 100% increase in ongoing cases, were NHS Fife, Lothian, Borders, Ayrshire and Arran, and Dumfries and Galloway. By the end of 2023, over 50% of referrals in all regions (except NHS Western Isles and NHS Shetland) were waiting for more than 12 weeks. Among specialties, those most severely impacted, with over 60% of inpatient cases waiting for more than 12 weeks, included Anaesthetics, Cardiothoracic Surgery, Community Dental Practice, ENT, General Surgery, Gynaecology, Neurosurgery, Oral and Maxillofacial Surgery, Paediatric Dentistry, Paediatric Surgery, Plastic Surgery, Trauma & Orthopaedics, and Urology. See Supplementary Materials for detailed analyses of inpatients by region (Supplementary Materials, Section 8) and by specialty (Supplementary Materials, Section 9).

Similarly, there were notable differences across regions and specialties in outpatient waiting times. The worst-affected health boards, with over a 100% increase in ongoing cases, were NHS Borders, Fife, and Lanarkshire. By the end of 2023, over 50% of referrals in most regions were waiting for more than 12 weeks. Among outpatient specialties, those with the highest proportion of cases waiting for more than 12 weeks (over 60%) included Dermatology, ENT, General Surgery, Gynaecology, Neurology, Ophthalmology, Oral Medicine, Oral and Maxillofacial Surgery, and Urology. See Supplementary Materials for detailed analyses of outpatients by region (Supplementary Materials, Section 10) and by specialty (Supplementary Materials, Section 11).

### Assessment of progress against the NHS recovery plan 2021–2026

**Fig. 2:**
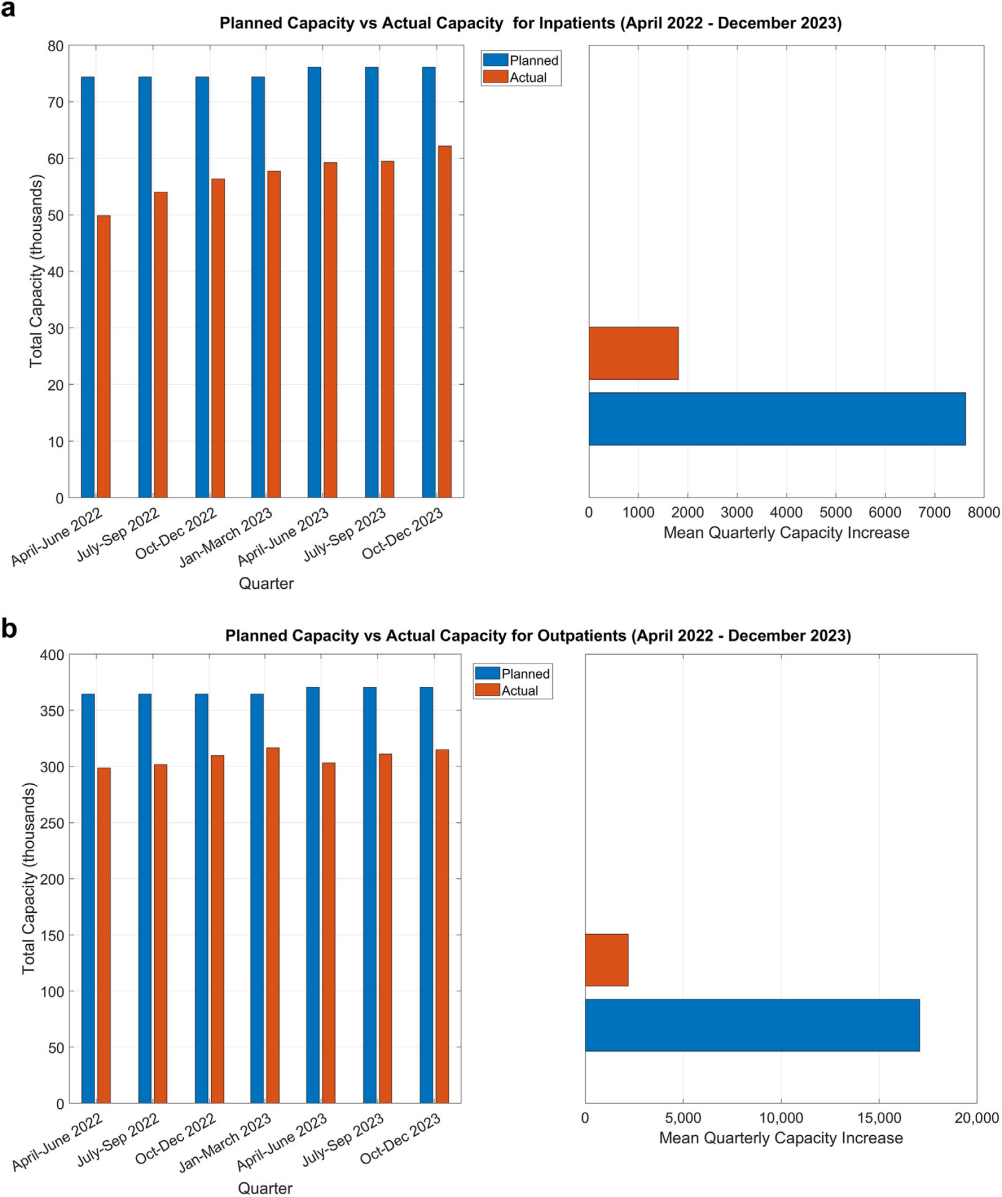
Quarterly recovery targets for capacity set by the Scottish government (in blue) based on the plans published in August 2021, and the observed capacity (in red) during the same period (April 2022–December 2023) for (a) inpatients, and (b) outpatients. The left panel shows the total capacity in each of the 7 quarters, and the right panel shows the mean quarterly increase.

The capacity observed in the quarter April–June 2022 was 49,862 (33% shortfall) for inpatients and 298,568 for outpatients (18% shortfall). The observed per quarter increase in capacity from April 2022 to December 2023 (seven quarters) was 1811 for inpatients (76% shortfall) and 2193 for outpatients (87% shortfall). Fig. 2 provides the target and the observed capacity in the period April 2022–December 2023 (see Supplementary Materials, Section 12 for further details).

## Discussion

There were year-on-year increases in the numbers of people waiting for elective procedures in Scotland since 2013, which was then compounded by the pandemic leading to over 680,000 cases by December 31, 2023. This worsening trend in the pre-pandemic period likely reflects broader systemic challenges, including the impact of austerity and underfunding in the NHS from the early 2010s onward, which have been highlighted in other research.^31^ While a single patient can have multiple concurrent referrals (for different, unrelated clinical pathways), most of these pending referrals are likely to be unique individuals; this therefore means that about 10% of the entire population in Scotland was waiting for elective care. These backlogs were seen across all health boards and specialities and will require substantial increased capacity sustained over several years to eliminate.

To our knowledge, this is the most comprehensive study to date in terms of geographical coverage (country-wide), time (longitudinal data over 11 years covering several years before pandemic and during pandemic), and stratification (by regions, specialties, and type of elective appointment). No prior study has combined as detailed an assessment of pandemic disruption on elective waiting lists, an evaluation of recovery efforts, and a statistical modelling approach to estimate the capacity increase required to address the backlog.

The scope of this paper is to quantify how much capacity increase would be needed to address the backlog, without making any further assumptions about how this increase will be achieved. The definition of capacity used in this study reflects the total throughput of the system, including all patients removed from the waiting list for any reason, whether treated or otherwise. While alternative definitions may focus solely on treated patients or available resources, we adopt a broader, system-level view to capture the real-world dynamics of patient flow. This approach is consistent with the data available.

A key challenge that affects the accuracy of our models is the unpredictability of future demand. We assumed a fixed quarterly demand over the three-year projection period and did not account for any potential external factors that might affect demand, such as public health crises or policy changes. However, the processed data and the accompanying code are publicly available for anyone to reproduce our findings and generate new projections under different sets of assumptions (Supplementary Materials, Section 13). This allows policymakers to explore different scenarios tailored to their specific questions.

Furthermore, we acknowledge that increasing capacity alone may not fully address the waiting list backlog. A multifaceted approach is needed, combining capacity increases with other measures such as improving waiting list management, promoting preventive healthcare, and workforce strategies aimed at recruitment and retention. Digital health solutions, such as telemedicine, may help manage less urgent cases, while innovative scheduling practices could optimise resource use. Moreover, strengthening primary and community care services may help prevent elective cases from escalating to urgent care needs. Comparisons between regions should be interpreted with caution, given variation in the proportion of the aging population across regions (with fewer over 65s living in cities compared to rural areas). Our figures on waiting lists do not correct for any differences in population demographics across regions. Lastly, our study relies on existing data reported by the various healthcare providers and may not capture the nuances of specific challenges faced by different health boards and specialties. Further research involving healthcare professionals and patients could help provide a complementary view to understand the factors behind the shortfall in recovery efforts, and the specific areas that need attention to ensure that the necessary capacity increase is realised. It is important to note that while our analysis focuses on the time patients wait for their first appointment, this does not capture the entire patient journey, including potential follow-up appointments. The metric we use measures entry into the care pathway, with follow-up visits considered part of the same initial case. While this approach provides an indication of system performance, it does not account for the complexity and duration of cases that require ongoing care. Further, outpatient and inpatient demand are modelled independently in this study, with no direct interaction between the two. While an increase in outpatient throughput may influence inpatient demand, estimating this interaction would require additional data, which is beyond the scope of the current analysis.

Several countries studying specific specialties, or covering a small geographical area reported a substantial impact during the early phases of the pandemic.^1^, ^2^, ^3^, ^4^, ^5^, ^6^, ^7^ Our study confirm those findings and further shows that the impact is substantial and widespread (country-wide, across specialties, across regions, and long-term). The study by Ghoshal et al. looked at data from the Massachusetts General Hospital, US and found that the pre-pandemic capacity was not restored in several specialties until the end of 2021.^32^ Our study shows that capacity in Scotland had not been restored by the end of 2023. Our modelling suggesting the need for a substantial increase in capacity to address the backlog aligns with recommendations from previous research.^25^^,^^33^ Our prior work suggested at least a 10% capacity increase to address the backlog in elective care in England,^25^ while Howlett et al. developed a projection model highlighting the long-term challenges faced by NHS England in dealing with the pandemic-induced backlog.^33^ The current study with a longer follow-up shows that the situation has worsened and a substantial increase in capacity is urgently needed to address the backlog. The comparison between England and Scotland provided in the Supplementary Material (Table S15, Supplementary Materials, Section 14) suggests a similar pattern of backlog growth in both countries during the pre-pandemic and pandemic periods. The previous studies from several countries and the comparison between England and Scotland further reinforces the notion that the challenges faced by NHS Scotland are not unique and reflect broader global trends within healthcare systems.

Addressing the substantial backlog is crucial to mitigate short-term harm and build long-term resilience, reducing the risk of similar situations in the future. Additionally, delays in elective care may place increased pressure on emergency services, compounding demand for urgent healthcare. Further, the backlog has broader implications for population health, including issues of equity. Patients from lower socioeconomic backgrounds may experience longer waits or reduced access to services, exacerbating health inequalities. Prioritising the most urgent cases, while ensuring equitable access, is critical in mitigating these effects. Recovery efforts to date have been inadequate. Collaboration between NHS health boards and the Scottish Government is essential for setting realistic recovery plans and ensuring sufficient funding is made available to allow for their effective implementation. Our work underscores the critical role of proactive health system monitoring. Such an oversight allows for continuous assessment of system resilience during and after emergencies. It also facilitates timely evaluation of recovery efforts, enabling course correction as needed. By highlighting these vulnerabilities, our study emphasises the urgent need to cultivate robust system resilience against future emergencies. This proactive approach is essential to mitigate the risk of a similar situation recurring and compromising patient care.

In conclusion, addressing the substantial backlog in elective care is crucial, but it must be approached as part of a broader strategy to enhance health system resilience. This includes improving equity in access to care, managing future demand, and optimising the use of existing resources. Only through a combination of capacity increases and systemic reforms can we mitigate the long-term effects of the pandemic and better prepare for future healthcare challenges.

## Contributors

AS conceived the analysis and oversaw all aspects of the study. SAS, with help from AS, CR, and KJ, designed the study. SAS conducted the analysis and wrote the first draft of the manuscript. SAS and KJ directly accessed and verified the underlying data. All authors edited the manuscript, had full access to all the data in this manuscript, and had final responsibility for the decision to submit for publication.

## Data sharing statement

All dataset used in this manuscript is derived from the ‘Stage of Treatment’ dataset that is publicly available from https://www.opendata.nhs.scot/dataset/stage-of-treatment-waiting-times under the UK open government license (https://www.nationalarchives.gov.uk/doc/open-government-licence/version/3/). The raw data and the processed data are also made available at https://github.com/syedahmar/ElectiveCare-Scotland under the UK open government licence.

## Declaration of interests

AS was an adviser to UK and Scottish Government COVID-19 advisory groups and is a member of Scottish Government's Standing Committee on Pandemic Preparedness. CR was an adviser to several Scottish and UK Government COVID-19 advisory groups; and is recipient of grants from Public Health Scotland, the Medical Research Council, Chief Scientist Office, UK Health Security Agency, and the National Institute for Health and Care Research. SAS and KJ declare no competing interests.
