# Supplementary material for: Impact of COVID-19 pandemic on elective care backlog trends, recovery efforts, and capacity needs to address backlogs in Scotland (2013–2023): a descriptive analysis and modelling study
Source: Lancet Reg Health Eur. 2025 Jan 9;50:101188. doi: 10.1016/j.lanepe.2024.101188 (PMC11910793; doi:10.1016/j.lanepe.2024.101188)
Supplement: Supplementary Material [file mmc1.pdf]

Supplementary Materials

Impact of COVID-19 pandemic on elective care backlog trends, recovery efforts, and capacity needs to address backlogs in Scotland (2013–2023): a descriptive analysis and modelling study

Syed Ahmar Shah[1], Karen Jeffrey[1], Chris Robertson[2], Aziz Sheikh[1,3]

[1]Usher Institute, The University of Edinburgh, Edinburgh, UK
[2] Department of Mathematics and Statistics, University of Strathclyde, Glasgow, UK
[3] Nuffield Department of Primary Care Health Sciences, University of Oxford, UK

Contents

1: Data flow diagram 2
2: Accounting for seasonality during estimation of quarterly mean 2
3: List of territorial Health Boards, and specialties 2
4: Model development and projections 4
    A: Import Dataset and undertake appropriate preprocessing 5
    B: Define different VARX models and estimate the model parameters 5
    C: Choose the 'optimal' model based on Akaike Information Criterion (AIC) 6
    D: Estimate capacity needs for backlog reduction using the optimal model 8
    E: Model parameters: Comparison with our previous work from England 9
5: Number of referrals waiting and projections 11
    Inpatients 11
    Outpatients 12
6: Waiting time distribution of completed cases 13
    Overall 14
    Inpatients 14
    Outpatients 15
7: Waiting time distribution of pending cases 16
    Overall 16
    Inpatients 17
    Outpatients 17
8: Distribution of pending cases stratified by NHS Health Board (Inpatients) 18
9: Distribution of pending cases stratified by Specialty (Inpatients) 21
10: Distribution of pending cases stratified by NHS Health Board (Outpatients) 24
11: Distribution of pending cases stratified by Specialty (Outpatients) 27
12: Assessment of progress against the Government's NHS Recovery Plan 31
    Inpatients 31
    Outpatients 32
13: Description of the code repository 33
14: Comparison of Scotland with England 35

Supplementary Materials

1: Data flow diagram

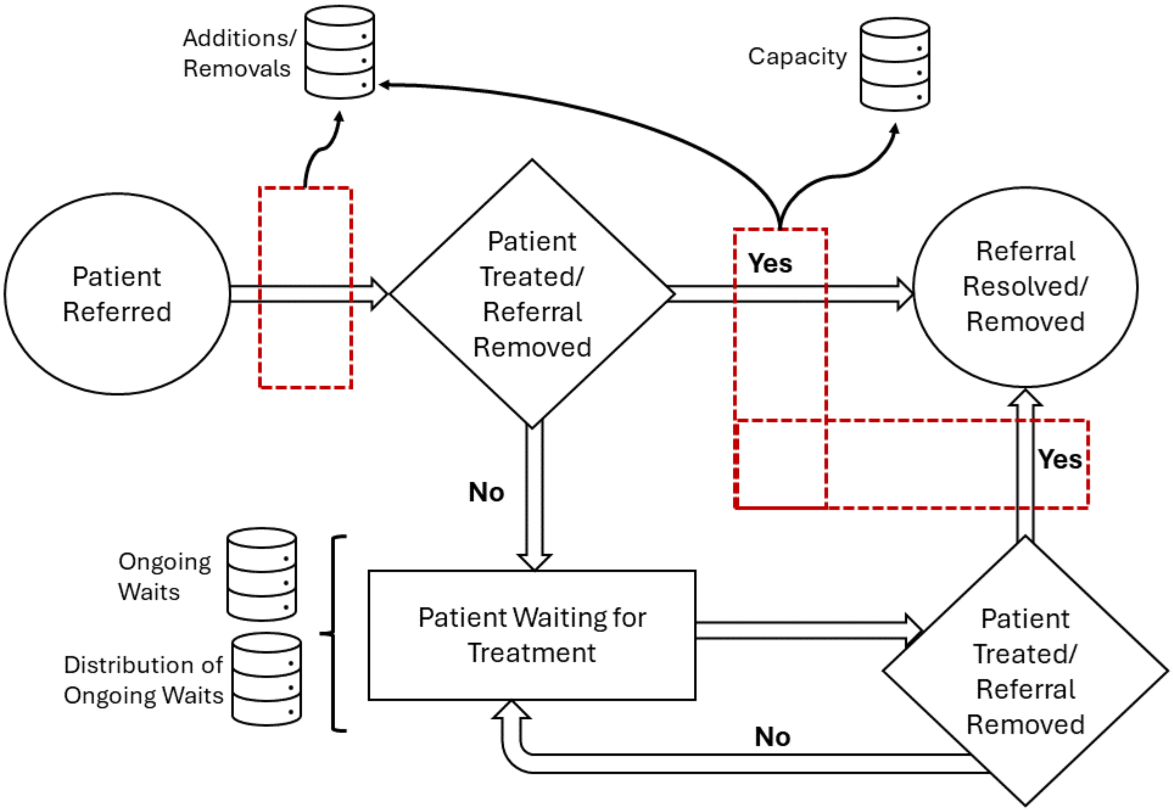

Figure S1: Conceptual diagram to show the various databases that were accessed in this study: additions/removals; capacity (referrals resolved/removed); ongoing waits; distribution of ongoing waits.

2: Accounting for seasonality during estimation of quarterly mean

To correctly estimate the 95% CI of quarterly mean for a given year, we need to account for any seasonality effect that might be present. In this study, we used the quarterly values of the three years we are interested in (2013, 2019, and 2023) giving us 12 data points and then fitted a linear model (y ~ year + season) without the intercept term while using both year and season as factors. The R code with the relevant function and an example use with a representative 12 sample time-series is provided in the associated GitHub repository ('GetCI_Adjust_Seasonality.R' at https://github.com/syedahmar/ElectiveCare-Scotland).

3: List of territorial Health Boards, and specialties

Table S1: List of 14 regions covering the entire Scotland, each associated with a territorial NHS board

Serial Number	Region
1	Ayrshire and Arran

Supplementary Materials

**2**	Borders
**3**	Dumfries and Galloway
**4**	Fife
**5**	Forth Valley
**6**	Grampian
**7**	Greater Glasgow and Clyde
**8**	Highland
**9**	Lanarkshire
**10**	Lothian
**11**	Orkney
**12**	Shetland
**13**	Tayside
**14**	Western Isles

Table S2: List of the 49 specialties and whether the data is available for either outpatients, inpatients/day cases or both.

**Serial Number**	**Specialty**	**Data Available for:**	
		**Outpatients**	**Inpatients/Day Cases**
**1**	Allergy	✓	X
**2**	Anaesthetics	✓	✓
**3**	Audiological Medicine	✓	X
**4**	Cardiac Surgery	✓	✓
**5**	Cardiology	✓	✓
**6**	Cardiothoracic Surgery	X	✓
**7**	Chemical Pathology	✓	X
**8**	Clinical Genetics	✓	X
**9**	Clinical Neurophysiology	✓	X
**10**	Clinical Oncology	✓	✓
**11**	Clinical Radiology	X	✓
**12**	Community Child Health	✓	X
**13**	Community Dental Practice	✓	✓
**14**	Dermatology	✓	✓
**15**	Diabetes	✓	X
**16**	Ear, Nose & Throat (ENT)	✓	✓
**17**	Endocrinology	✓	✓
**18**	Gastroenterology	✓	✓
**19**	General Medicine	✓	✓
**20**	General Surgery	✓	✓
**21**	General Surgery (excluding Vascular)	✓	✓
**22**	Geriatric Medicine	✓	X
**23**	Gynaecology	✓	✓
**24**	Haematology	✓	✓
**25**	Immunology	✓	X
**26**	Infectious Diseases	✓	X
**27**	Medical Oncology	✓	✓
**28**	Neurology	✓	✓

**29**	Neurosurgery	✓	✓
**30**	Ophthalmology	✓	✓
**31**	Oral Medicine	✓	X
**32**	Oral Surgery	✓	✓
**33**	Oral Maxillofacial Surgery	✓	✓
**34**	Orthodontics	✓	X
**35**	Paediatric Dentistry	✓	✓
**36**	Paediatric Surgery	✓	✓
**37**	Paediatrics	✓	✓
**38**	Pain Management	✓	✓
**39**	Palliative Medicine	✓	X
**40**	Plastic Surgery	✓	✓
**41**	Rehabilitation Medicine	✓	X
**42**	Renal Medicine	✓	✓
**43**	Respiratory Medicine	✓	✓
**44**	Restorative Dentistry	✓	X
**45**	Rheumatology	✓	✓
**46**	Thoracic Surgery	✓	✓
**47**	Trauma and Orthopaedic Surgery	✓	✓
**48**	Urology	✓	✓
**49**	Vascular Surgery	✓	✓
**Total Specialties for which data is available**		**47**	**34**

4: Model development and projections

Similar to our previous work,(https://doi.org/10.1016/S0140-6736(23)02744-7) our model can conceptually be represented as a stocks-and-flows system where the total number of referrals waiting (the 'stocks') depends on the quarterly demand (the incoming flow rate) and the quarterly outgoing rate (the outgoing flow rate) as illustrated in Figure S2.

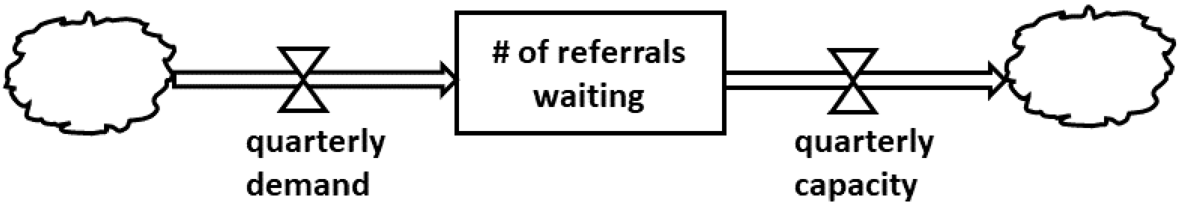

Figure S2: Stocks-and-Flows model illustrating how the number of people waiting (the stocks) is affected by the quarterly demand (incoming flow rate) and the quarterly capacity (the outgoing flow rate).

Our modelling approach consists of the following steps:

A. Import dataset and undertake appropriate preprocessing
B. Define different Vector Autoregressive with exogenous variables (VARX) models and estimate the model parameters
C. Choose the 'optimal' model based on Akaike Information Criterion (AIC)
D. Forecast the total number of pending patient referrals, the peak number and the corresponding time under different capacity increase scenarios

Supplementary Materials

A: Import Dataset and undertake appropriate preprocessing

The dataset we used to build the model consists of three time-series: total waiting; additions (termed demand in the conceptual model); removals (termed capacity in our conceptual model). For the waiting time-series, we used the 'Ongoing Waits' dataset which provides the total number of referrals waiting at the end of a given month. For the additions and removals, we used the "Additions and Removals" dataset that provides the total number of additions and removals at the end of a given quarter. Both these datasets are available from the "Stage of Treatment Waiting Times" available at https://www.opendata.nhs.scot/dataset/stage-of-treatment-waiting-times under the UK Open Government Licence (https://www.nationalarchives.gov.uk/doc/open-government-licence/version/3/).

After importing the datasets, we undertook some pre-processing to ensure that we have three time-series (waiting, capacity, and demand) that are sampled at the same rate (quarterly) and cover the period of interest (January 1, 2013 – December 31, 2023). For the pre-processing, we first down-sampled the monthly waiting time-series to make it quarterly and then selected the data that pertained to the study period. After the dataset import and the relevant pre-processing, the final dataset had 44 samples covering the period (January 1, 2013 – December 31, 2023).

B: Define different VARX models and estimate the model parameters

To implement the conceptual model using the real-world data, we have formulated it as a vector autoregression with exogenous variables (VARX) model where the number of referrals waiting is the response time series, and the time-series associated with demand and capacity are the exogenous variables. (Wei WW. Multivariate time series analysis and applications. John Wiley & Sons; 2018 Dec 31)

In a perfectly closed system, the number of people waiting would have directly corresponded to the number of referrals received and inversely corresponded to the number of referrals addressed (deemed to be the capacity of the system). In this scenario, a simple stock and flow model would be sufficient. This is a special case of a VARX model where the coefficients for exogenous time series are predetermined to be +1 and -1. However, the real-world situation we're modelling is not a closed system. The number waiting is also influenced by additional factors such as deaths, hospital clinicians declining referrals, patients removing themselves from the list for private care, and so on.

To account for these extraneous factors not captured by a simple stock and flow model, we employed a VARX model. The coefficients in this model are determined based on the actual data, rather than being fixed a priori at +1 and -1. It's important to note that "removals" (considered the "capacity" in this study) encompass all referrals removed from the system within a quarter. This includes those who receive treatment (the majority) but also those removed for various reasons like being referred back to primary care, transferred to a different specialty, or no longer requiring treatment. Therefore, in this study, "capacity" refers to the total number of referrals removed from the system in a given quarter, regardless of the reason for removal.

To identify the most appropriate model and the associated parameters, we used two variations of the VARX model as represented by the following equation:

Supplementary Materials

$$\boldsymbol{y_w[n] = c + \sum_{j=1}^{p} \Phi_j\, y_w[n-j] + \beta_d x_d[n] + \beta_c x_c[n] + \varepsilon_n} \qquad \textbf{(1)}$$ where

n: sequence number (discrete-time equivalent of time variable)
$y_w[n]$: number of pending patient referrals at the end of quarter *n*
c: constant offset in the model
$y_w[n-j]$: number of pending patient referrals at the end of quarter *n-j*
p: model order (number of lags in the response time series)
Φ_j: *1xp* vector of autoregressive coefficients
$x_d[n]$: quarterly demand in quarter *n* (an exogenous time-series)
β_d: regression coefficient associated with the quarterly demand time-series
$x_c[n]$: quarterly capacity in quarter *n* (an exogenous time-series)
β_c: regression coefficient associated with the quarterly capacity time-series
ε_n: the error term (white noise) with a mean of 0 and constant variance

The two variations of the VARX model we considered were: model with *p=4* (seasonality accounted for, but a complex model that also includes lag terms of 2, and 3); model with *p=4* but using only lag of 1 and 4 (a more parsimonious version where seasonality is accounted for, but the lag terms of 2 and 3 are not included in the model). Note that the main parameter that lends itself to this kind of sensitivity analysis is the lag structure. Other parameters, such as the intercept, inflow, outflow, and error terms, are intrinsic to the model and were not suitable for similar exploration.

We first developed a model for all elective types, and then repeated the same analysis separately for inpatients, and outpatients. All the implementations were undertaken in MATLAB. To create a VARX object, we used the varx function. To find the most appropriate values of each parameter, we used the estimate function which uses maximum likelihood to determine the best fit values. The Matlab script for finding the model parameter is “GetModelParameters_final.m” available in the project GitHub repository.

C: Choose the ‘optimal’ model based on Akaike Information Criterion (AIC)

The AIC is a well-known information criterion used for model selection. However, in scenarios with small sample sizes, AIC can exhibit a tendency towards overfitting. This occurs when models with excessive complexity are favoured, leading to poor generalisability on unseen data. The AICc (Akaike Information Criterion with correction) addresses this limitation by incorporating a penalty term that scales with the number of model parameters (k) and the sample size (n). This penalty term discourages models with excessive complexity, particularly in settings where n/k is relatively small (less than 40, as suggested previously (Burnham, K.P. and Anderson, D.R. eds., 2002. *Model selection and multimodel inference: a practical information-theoretic approach*. New York, NY: Springer New York.)). As the sample size increases, the penalty term in AICc diminishes, asymptotically converging towards the original AIC formulation. In our study, n/k is <40 and we have therefore computed the AICc for model comparison. We selected the model with the lost AICc to be the ‘optimal’ model. For completeness, we also computed and reported the AIC, the Bayesian Information Criterion (BIC), and the log likelihood.

Tables-S3-S5 provide the model equation, the estimated parameters and the associated log likelihood, AIC, and BIC for all elective types, inpatients, and outpatients respectively. In

each table, the row with the least AIC (corresponding to the optimal model) is highlighted in bold. In all the three cases, the optimal model is the VARX model with a lag of 1 and 4. This 'optimal' order was subsequently used for projections.

Table S3: Different VARX models for all elective types, the associated equation, and the AIC, BIC, log likelihood. Note that the aggregate data (across Scotland, covering all specialties) were missing for the period covering April 1, 2017 – December 31, 2018 (i.e. 7 quarterly data points) thereby giving us a total of 37 quarterly data points to estimate the model parameters

**VARX Model with a lag of 1 and 4 only**	
Model Equation	$y_w[n] = c + \Phi_1 y_w[n-1] + \Phi_4 y_w[n-4] + \beta_d x_d[n] + \beta_c x_c[n] + \varepsilon_n$
Estimated Parameters (standard error)	c: 230.801 (10040.742); Φ_1: 0.965 (0.030); Φ_4: 0.040 (0.037); β_d: 0.958 (0.064); β_c: -0.957 (0.058); ε_n: 5906.945
Log Likelihood	-333.393
AIC	676.786
**AICc**	**679.009** (*n* (sample size) = 33; *k* (number of parameters) = 5)
BIC	684.269
**VARX Model with lags of 1 to 4**	
Model Equation	$y_w[n] = c + \Phi_1 y_w[n-1] + \Phi_2 y_w[n-2] + \Phi_3 y_w[n-3] + \Phi_4 y_w[n-4] + \beta_d x_d[n] + \beta_c x_c[n] + \varepsilon_n$
Estimated Parameters (standard error)	c: 5599.967 (10048.116); Φ_1: 1.098 (0.099); Φ_2: -0.277 (0.174)); Φ_3: 0.284 (0.157); Φ_4: -0.103 (0.087); β_d: 0.864 (0.086); β_c: -0.874 (0.079); ε_n: 5631.842
Log Likelihood	-331.819
AIC	677.639
AICc	682.119 (*n* (sample size) = 33; *k* (number of parameters) = 7)
BIC	688.114

Table S4: Different VARX models for inpatients, the associated equation, and the AIC, BIC, log likelihood. Note that the aggregate data (across Scotland, covering all specialties) were missing for the period covering April 1, 2017 – June 30, 2018 (i.e. 5 quarterly data points) thereby giving us a total of 39 quarterly data points to estimate the model parameters

**VARX Model with a lag of 1 and 4 only**	
Model Equation	$y_w[n] = c + \Phi_1 y_w[n-1] + \Phi_4 y_w[n-4] + \beta_d x_d[n] + \beta_c x_c[n] + \varepsilon_n$
Estimated Parameters (standard error)	c: 779.275 (2007.835); Φ_1: 1.000 (0.035); Φ_4: -0.007 (0.040); β_d: 0.998 (0.096); β_c: -0.998 (0.087); ε_n: 1480.242
Log Likelihood	-305.161
AIC	620.323
**AICc**	**622.392** (*n* (sample size) = 35; *k* (number of parameters) = 5)
BIC	628.100
**VARX Model with lags of 1 to 4**	
Model Equation	$y_w[n] = c + \Phi_1 y_w[n-1] + \Phi_2 y_w[n-2] + \Phi_3 y_w[n-3] + \Phi_4 y_w[n-4] + \beta_d x_d[n] + \beta_c x_c[n] + \varepsilon_n$
Estimated Parameters (standard error)	c: 841.543 (2015.198); Φ_1: 1.022 (0.082); Φ_2: -0.047 (0.134); Φ_3: 0.036 (0.131); Φ_4: -0.018 (0.083); β_d: 0.989 (0.100); β_c: -0.990 (0.091); ε_n: 1477.602
Log Likelihood	-305.099
AIC	624.198
AICc	628.346 (*n* (sample size) = 35; *k* (number of parameters) = 7)
BIC	635.085

Table S5: Different VARX models for outpatients, the associated equation, and the AIC, BIC, log likelihood. Note that the aggregate data (across Scotland, covering all specialties) were missing for the period covering April 1, 2017 – December 31, 2018 (i.e. 7 quarterly data points) thereby giving us a total of 37 quarterly data points to estimate the model parameters

**VARX Model with a lag of 1 and 4 only**	
Model Equation	$y_w[n] = c + \Phi_1 y_w[n-1] + \Phi_4 y_w[n-4] + \beta_d x_d[n] + \beta_c x_c[n] + \varepsilon_n$
Estimated Parameters (standard error)	c: 1128.491 (6392.444); Φ_1: 0.971 (0.020); Φ_4: 0.033 (0.025); β_d: 0.976 (0.040); β_c: -0.977 (0.036); ε_n: 3729.532

Log Likelihood	-318.218
AIC	646.436
**AICc**	**648.659** (*n* (sample size) = 33; *k* (number of parameters) = 5)
BIC	653.919
**VARX Model with lags of 1 to 4**	
Model Equation	$y_w[n] = c + \Phi_1 y_w[n-1] + \Phi_2 y_w[n-2] + \Phi_3 y_w[n-3] + \Phi_4 y_w[n-4] + \beta_d x_d[n] + \beta_c x_c[n] + \varepsilon_n$
Estimated Parameters (standard error)	c: 3470.523 (6327.025); Φ_1: 1.033 (0.058); Φ_2: -0.136 (0.103); Φ_3: 0.152 (0.095); Φ_4: -0.046 (0.054); β_d: 0.930 (0.052); β_c: -0.937 (0.048); ε_n: 3587.302
Log Likelihood	-316.935
AIC	647.870
AICc	652.350 (*n* (sample size) = 33; *k* (number of parameters) = 7)
BIC	658.346

D: Estimate capacity needs for backlog reduction using the optimal model

We estimated the number of pending referrals over the next three years (January 1, 2024 – December 31, 2026, the projection period) under multiple scenarios where the healthcare elective capacity was increased from 0-25% in increments of 5% and the starting capacity was taken to be the mean quarterly capacity in 2023 (the latest year with complete data observed and reported).

In our simulations, we assumed the capacity to gradually increase at a constant rate during the projection period such that the total increase by the end of the 3-year period is x% where x varies from 0 to 25 (Figure S3 shows the capacity time-series for the various capacity-increase scenarios, separately for all elective types, inpatients, and outpatients). For the demand time-series, we assumed the demand to be equal to the mean quarterly demand in 2023. Figure S4 shows the demand time-series, separately for all elective types, inpatients, and outpatients.

For each elective type and each scenario, we ran 1,000 simulations and, in each simulation, iteratively computed the total pending referrals as a function of previous total pending referrals, current quarterly demand and capacity using the optimal model's parameters previously learned (in Step C). We subsequently computed the median and the 95% confidence intervals (2.5th and 97.5th percentiles) and plotted the resulting projections. The Matlab script "HealthcareDisruption_Projections_Scotland_final.m" available in the project GitHub repository contains the relevant code to undertake all the steps described in this section.

Supplementary Materials

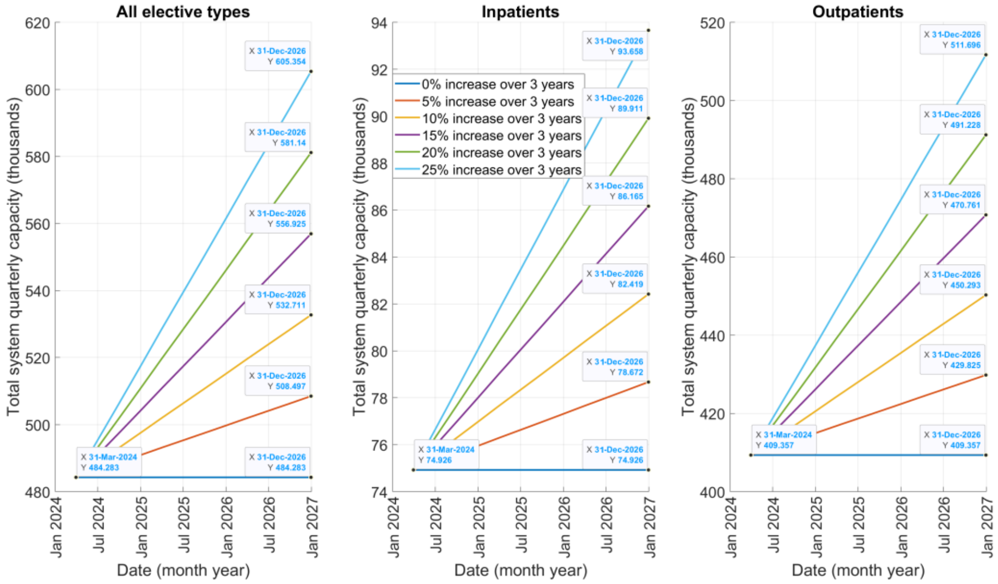

Figure S3: The assumed quarterly capacity time-series assuming the capacity will increase x% over the three years (January 1, 2024 - December 31, 2026). Note that the first data point plotted at March 31, 2024 corresponds to the first quarter of 2024 (January 1, 2024 – March 31, 2024)

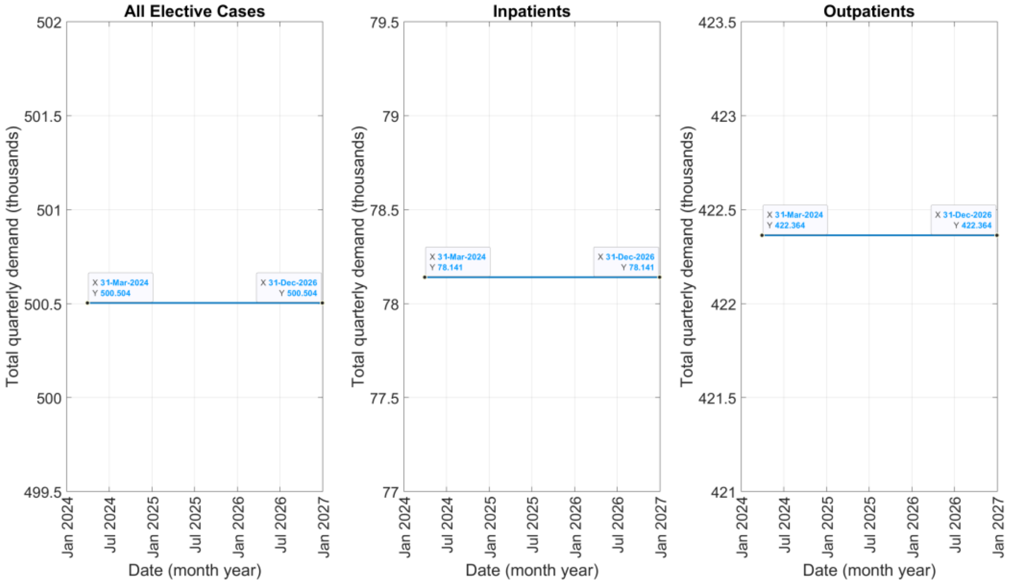

Figure S4: The assumed quarterly demand time-series assuming the demand will stay constant and equal to the mean demand observed in 2023. Note that the first data point plotted at March 31, 2024 corresponds to the first quarter of 2024 (January 1, 2024 – March 31, 2024)

E: Model parameters: Comparison with our previous work from England

In all healthcare systems, we can reasonably expect that most of the people added to the system (i.e. waiting list) will be removed because they got treated. However, a small proportion of referrals would be removed due to other factors such as transferred, referred back to primary care, treatment no longer required, or death. Regardless of the reasons for how patients are removed from the waiting list, our statistical model includes one explicit term for outflow and one explicit term for inflow. By learning the values of these two parameters, this approach

models any healthcare system with one input and one output exogenous time-series where the coefficients learned reflect the total inflow and outflow dynamics of the healthcare system.

In our previous study using data from England,(https://doi.org/10.1016/S0140-6736(23)02744-7) we only had access to total treated when fitting the model (i.e. the exogeneous time-series pertaining to capacity in that work referred to treated only). The learned values of the coefficients in the model were therefore much different than a simple stocks-and-flows model (i.e. a model with a +1 for demand and a - 1 for capacity), to account for the proportion of referrals that were removed for reasons other than treatment.

The 'Additions/Removals' dataset from Scotland does include a time-series for treated but it also includes a time-series for total removed. In our modelling in this study, we have used the 'capacity' to mean any referral removed (most of which is because they are treated but it also includes any other reasons for being removed from the system, see Figure S5 for illustration). Since the outflow time-series used in this study already now includes any removal from system including going private, transferred, treatment denied, death, etc., the learned parameters are close to a magnitude of 1 (like a simple stocks-and-flows model).

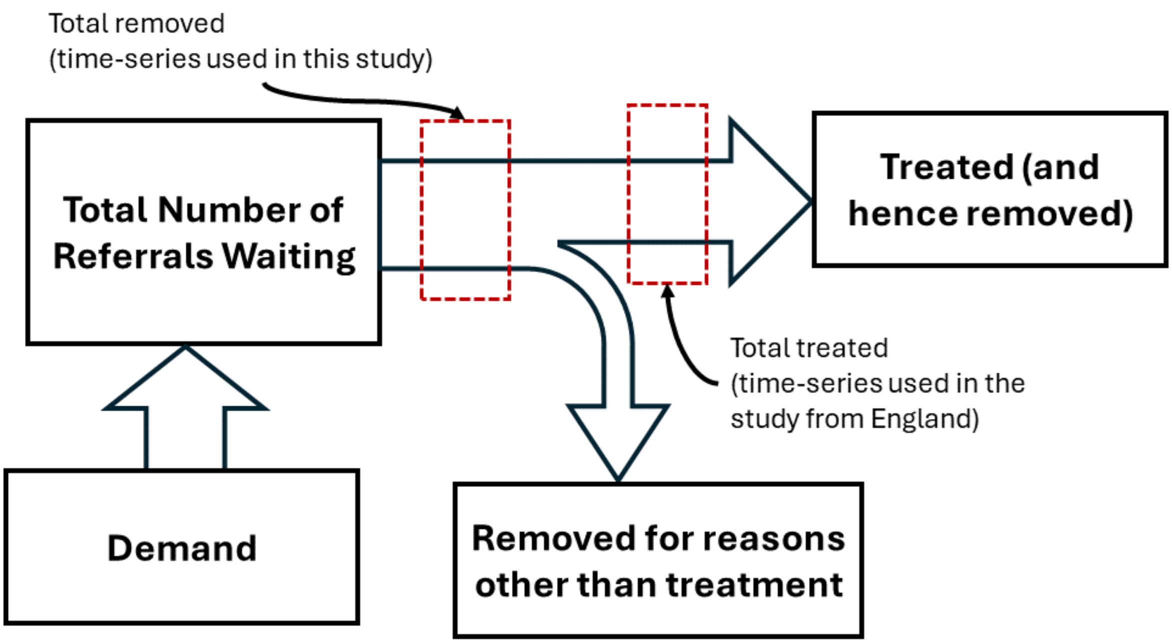

Figure S5: The total number of pending referrals at any given time is dependent on the total inflow (demand) and outflow (removed due to treatment, or due to other reasons such as treatment denied, referred back to primary care, going private, death, etc.). In this study, we used the total removed as the capacity time-series. In our previous study from England, only the total treated time-series was available. The learned coefficients of the inflow/outflow are able to model both cases

Our flexible model works in either case: whether we only use total treated (as was the case in England because the total removed was not available) or whether we use the total removed if available (as is the case in Scotland). The important point is to ensure that during projections, the same definition of capacity is used. In this study, our baseline capacity (to use during the projections period) was based on quarterly removed in the period when data was recorded. In our previous work from England,(https://doi.org/10.1016/S0140-6736(23)02744-7) our baseline capacity used in projections was based on monthly treated during the period when the data was available.

Supplementary Materials

To further illustrate this point, we have repeated the modelling for inpatients in Scotland but used the total treated time-series this time. The estimated coefficient of capacity, β_c, in this case is -1.245 while the coefficient for demand, β_d, is 1.090. The estimated coefficient of capacity is now substantially different than what we found when we used the total removed as the capacity time-series (see Table S6 where the parameters estimated used total treated only as the capacity time-series and compare with Table S4 where the capacity time-series was the total removed i.e. treated or removed for any other reason). The difference between the estimated coefficients for demand and capacity suggests that approximately 25% of referrals are removed from the system for reasons other than due to treatment.

Table S6: VARX model with a lag of 1 and 4 only, and with using the total treated time-series as capacity (as opposed to using the total removed time-series as capacity) to illustrate the difference it makes to the estimated parameters when the capacity time-series does not include any removals due to reasons other than treated

**VARX Model with a lag of 1 and 4 only and using quarterly treated as capacity (Inpatients)**	
Model Equation	$y_w[n] = c + \Phi_1 y_w[n-1] + \Phi_4 y_w[n-4] + \beta_d x_d[n] + \beta_c x_c[n] + \varepsilon_n$
Estimated Parameters (standard error)	c: -1746.849 (1825.797); Φ_1: 0.950 (0.032); Φ_4: 0.008 (0.035); β_d: 1.090 (0.089); β_c: -1.245 (0.093); ε_n: 1297.016
Log Likelihood	-300.537
AIC	611.073
AICc	613.142 (*n* (sample size) = 35; *k* (number of parameters) = 5)
BIC	618.850

5: Number of referrals waiting and projections

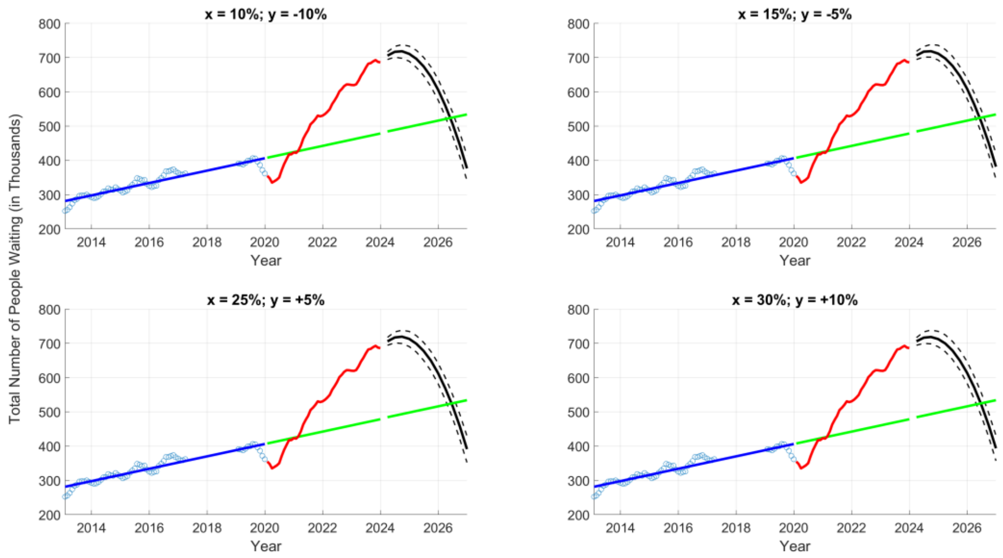

Figure S6: Number of Pending Cases (All Elective Types), showing the extent of disruption and projections under scenarios where the backlog created during the pandemic is eliminated by the end of the 3-year projection period, with capacity increasing linearly by x% and demand changing linearly by y%. The starting capacity and demand are based on the mean quarterly values from 2023, the most recent year with complete data.

Inpatients

Figure S7 shows the total number of pending appointments from January 1, 2013, to December 31, 2023, the projections for the next 3 years (January 1, 2024 – December 31, 2026) and the

counterfactual linear projections. The list grew by about 5,000 per year in the seven years prior to the pandemic (a growth of 34,418 -76% increase- from 45,532 on January 1, 2013, to 79,950 in December 2019). In the four years since then (January 1, 2020, to December 31, 2023), the waiting list has grown by about 19,000 per year (a growth of 75,080 – 94% increase – from 79,950 on January 1, 2020, to 155,030 on December 31, 2023).

Our projections model suggests that the total number waiting will peak at 165,093 by December 2025, 160,938 by December 2024, 159,527 by September 2024, 158,587 by June 2024 and 158,464 by June 2024 if the total capacity is increased by 5%, 10%, 15%, 20% and 25% implemented over the next three years (in reference to the mean capacity during January 1, 2023 – December 31, 2023), respectively. To remove only the backlog created during the pandemic (i.e. reach the pre-pandemic level) over the next three years, the capacity must increase by at least 25%. Without any increase in capacity, the pending referrals are predicted to reach 183,760 by December 2026.

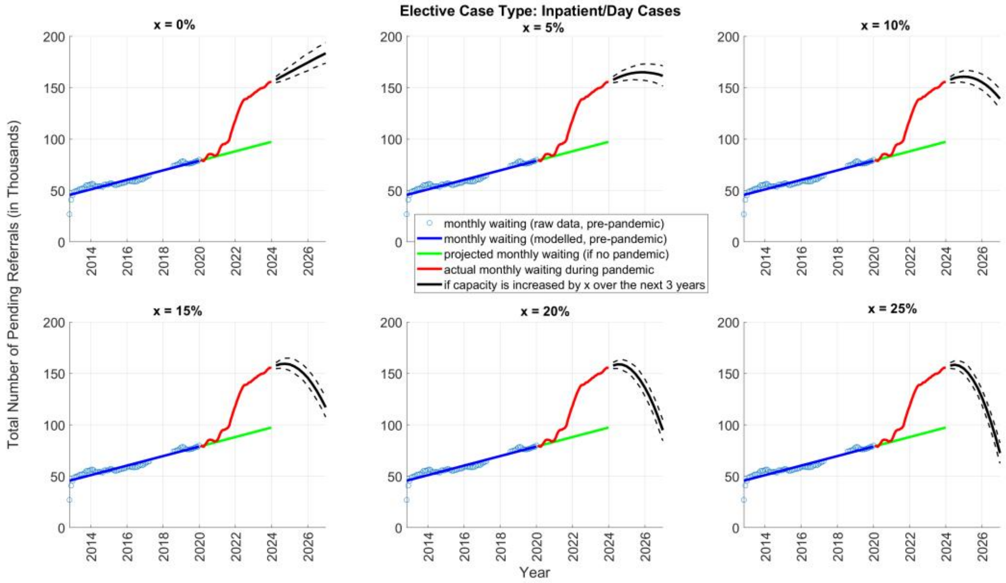

Figure S7: Number of Pending Inpatient cases showing the extent of disruption and the projections

Outpatients

Figure S8 shows the total number of pending appointments from January 1, 2013, to December 31, 2023, the projections for the next 3 years (January 1, 2024 – December 31, 2026) and the linear projections (the counterfactuals if the pandemic had not happened) based on the data from the pre-pandemic period (January 1, 2013, to December 31, 2019). The list grew by approximately 10,000 referrals/year in the seven years prior to the pandemic (a growth of 72,155 – a 35% increase – from 209,080 on January 1, 2013, to 281,235 on December 31, 2019). In the four years since then (January 1, 2020, to December 31, 2023) the waiting list grew by about 61,000/year (a growth of 248,086 – an 88% increase – from 281,235 on January 1, 2020, to 529,321 on December 31, 2023).

Our projections model suggests that the total number of patient referrals awaiting treatment will peak at 586,756 by December 2025, 559,986 by December 2024, 551,623 by September 2024, 546,841 by June 2024, and 545,033 by June 2024 if the total capacity over the next three

years is increased by 5%, 10%, 15%, 20% and 25% (in reference to the mean capacity during January 1, 2023 – December 31, 2023) respectively. To fully address the backlog created during the pandemic over the next three years (i.e. reach pre-pandemic levels), the capacity must increase by at least 20%. Without any increase in capacity, the total pending referrals are predicted to reach 689,476 by December 2026.

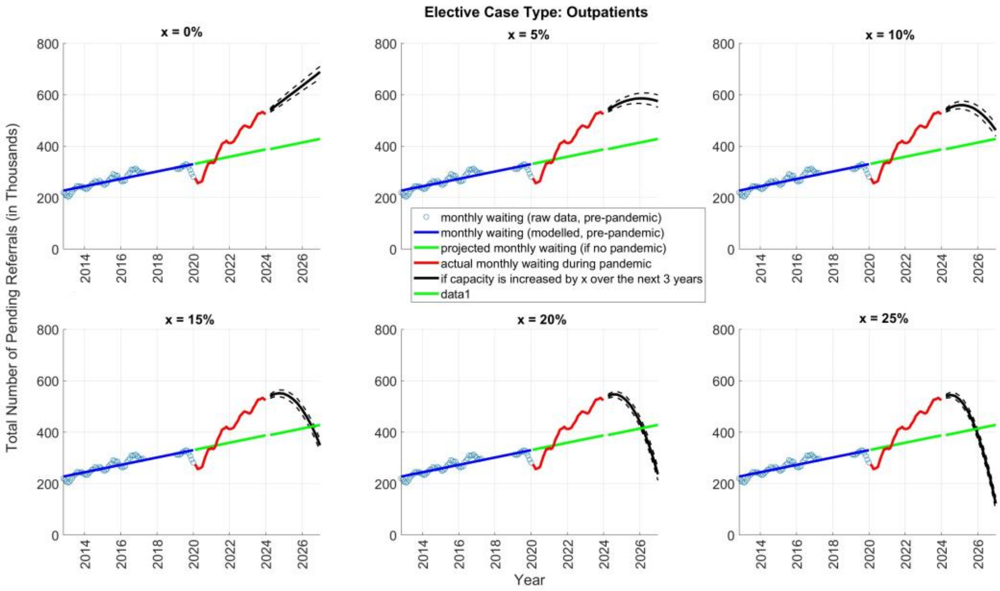

Figure S8: Number of Pending Outpatient Referrals showing the extent of disruption and the projections

6: Waiting time distribution of completed cases

For the waiting time distribution data of completed cases, the data was not available for Tayside in the period from April 2017 – December 2018. Consequently, we used cubic spline interpolation for the missing period when plotting the distribution across Scotland. The code that implements cubic spline interpolation is provided in "Plot_CompletedDistribution.m" (see section 13).

Supplementary Materials

Overall

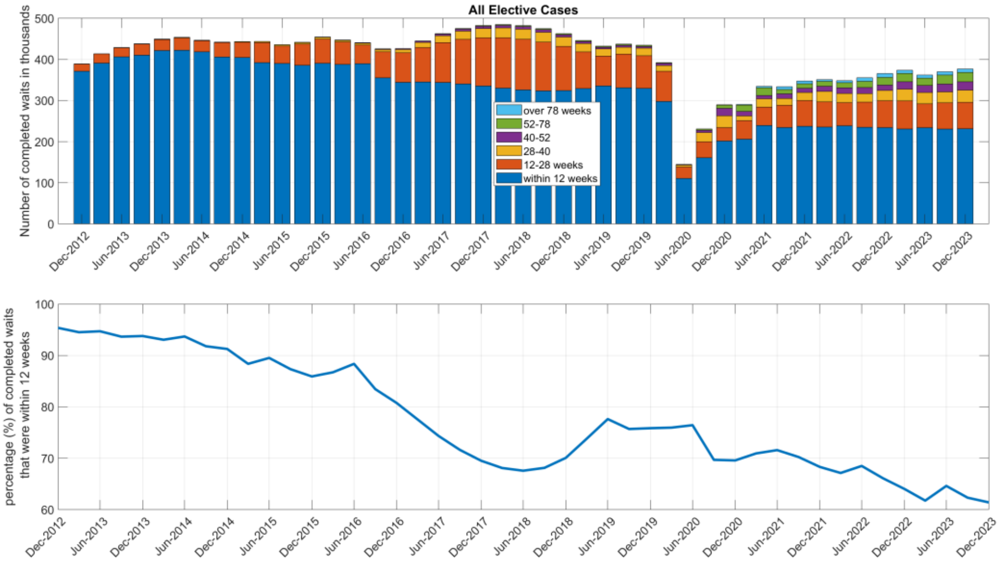

Figure S9: Distribution of completed cases (all elective types) in terms of how long before the patients were seen (upper panel) and the percentage of cases that were seen within 12 weeks (lower panel)

Inpatients

Figure S10 provides the distribution of completed cases and the percentage seen within 12 weeks. There were 79,858 inpatient appointments in the quarter January-March 2013, with most (78,408; 98%) seen within 12 weeks. This capacity gradually declined during the pre-pandemic period and in the quarter just before the pandemic (October – December 2019), there were 69,200 inpatient appointments. However, by this time, the proportion of those who had to wait for less than 12 weeks before being seen had dropped to 71%. Since then, there has been a substantial decline. During the early months of the pandemic (April – June 2020), the capacity was at the lowest point since December 2012 with only 14,677 inpatient appointments (a drop of 79% compared to October-December 2019). There was subsequent recovery, but the capacity has continued to remain below the pre-pandemic period. In the last quarter of the

study period (October-December 2023), there were 61,896 inpatient appointments with only 58% seen within 12 weeks.

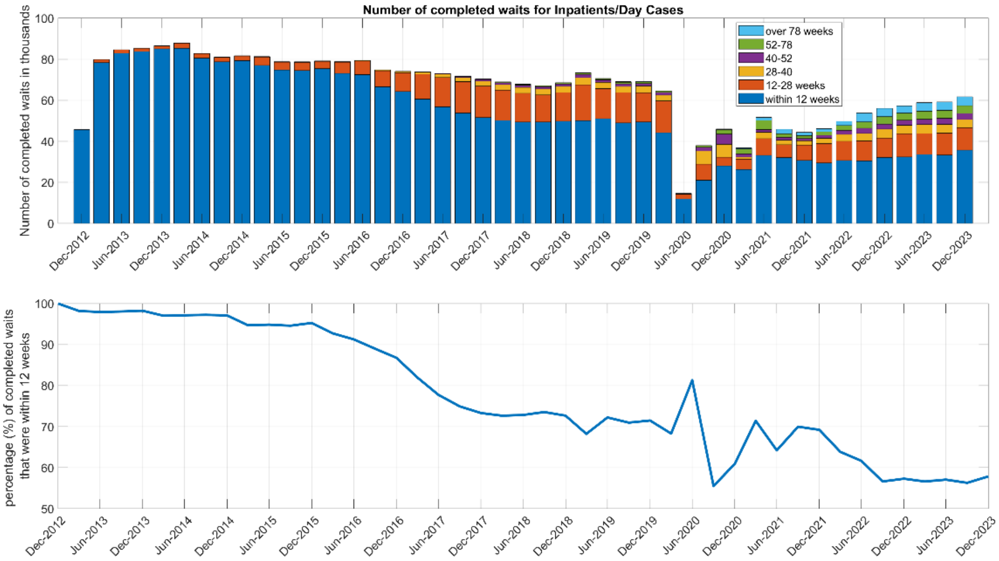

Figure S10: Distribution of completed inpatient/day cases in terms of how long before the patients were seen (upper panel) and the percentage of cases that were seen within 12 weeks (lower panel)

Outpatients

Figure S11 provides the distribution of completed cases and the percentage that were seen within 12 weeks of referral, from late 2012 until late 2023. There were 333,860 appointments in the quarter January-March 2013, with most (312,633; 94%) seen within 12 weeks. In the quarter just before the pandemic (October – December 2019), 365,415 outpatient appointments took place. However, by this time, the proportion of patients seen within 12 weeks of referral had fallen to 77%. Since the beginning of the pandemic, there has been a substantial decline in capacity. During the April–June 2020 quarter, the capacity was at the lowest point since December 2012 with 129,986 outpatients (a drop of 64% compared to October-December 2019). Since then, there has been some recovery, but the capacity has continued to remain below the pre-pandemic period. In the last quarter of the study period (October-December 2023), there were 314,910 appointments with 62% of patients seen within 12 weeks of referral.

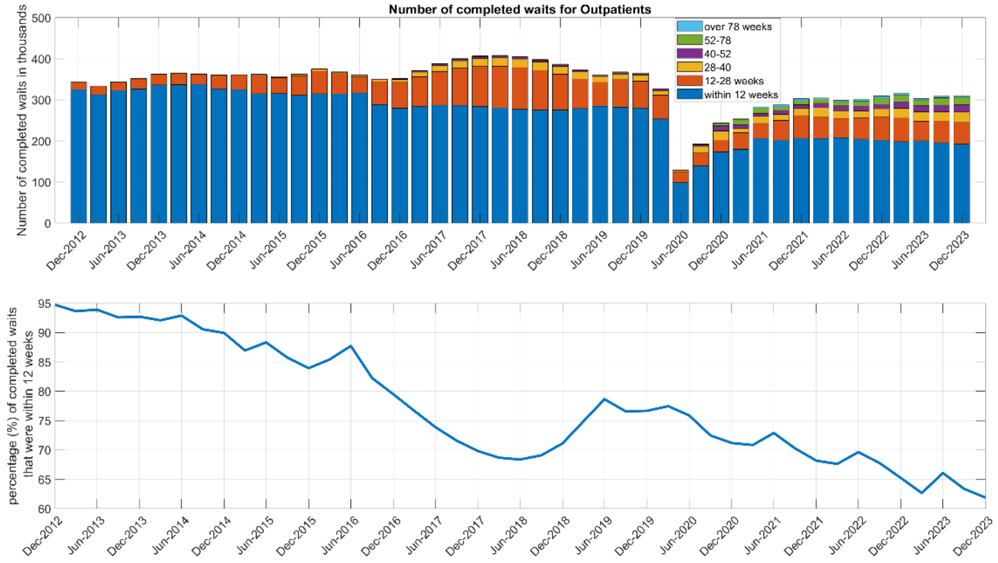

Figure S11: Distribution of completed outpatient cases in terms of how long before the patients were seen (upper panel) and the percentage of cases that were seen within 12 weeks (lower panel)

7: Waiting time distribution of pending cases

For the waiting time distribution data of pending cases, the data was not available for Tayside in the period from April 2017 – December 2018. Consequently, we used cubic spline interpolation for the missing period when plotting the distribution across Scotland. The code that implements cubic spline interpolation is provided in "Plot_OngoingDistribution.m" (see section 13).

Overall

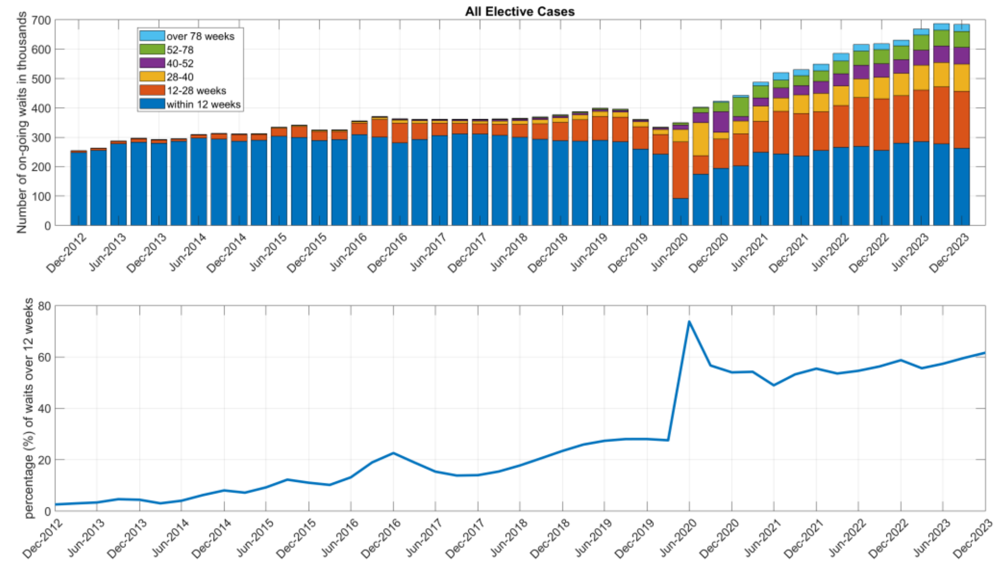

Figure S12: Distribution of ongoing cases (all elective types) in terms of how long the patients have been waiting for (upper panel) and the percentage of cases that have been waiting for over 12 weeks (lower panel)

Supplementary Materials

Inpatients

Figure S13 provides the distribution of ongoing cases and the percentage waiting for over 12 weeks. On January 1, 2013, only 0.1% patients (63 out of 45,532) were waiting for over 12 weeks. This percentage slowly grew and reached 32% on December 31, 2019 (25,980 out of 79,950). During the pandemic, the percentage of patients who waited for longer than 12 weeks grew substantially, reaching 68% on December 31, 2023 (104,769 out of 155,030).

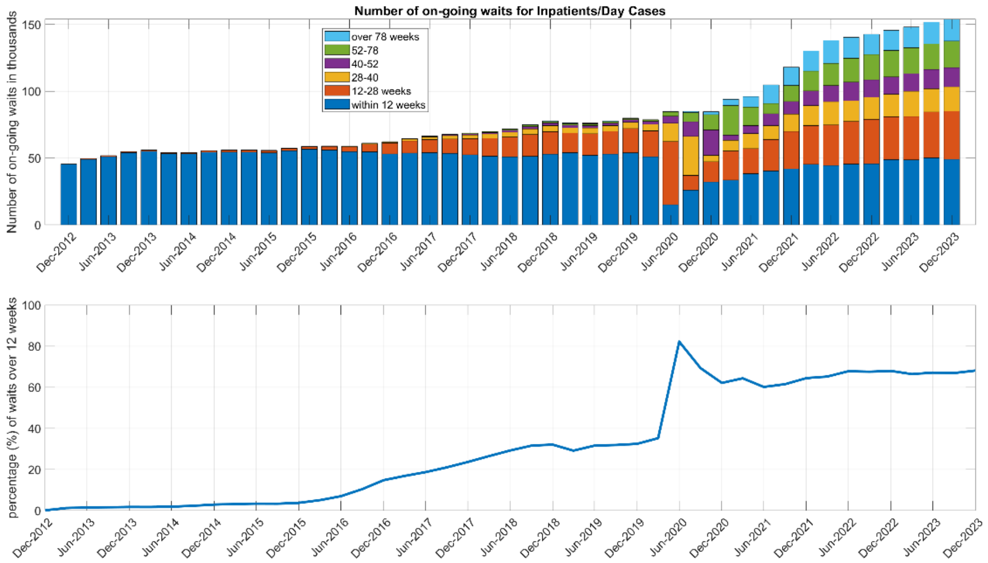

Figure S13: Distribution of ongoing inpatient cases in terms of how long the patients have been waiting for (upper panel) and the percentage of cases that have been waiting for over 12 weeks (lower panel)

Outpatients

Figure S14 presents the distribution of ongoing inpatient cases and the percentage that were waiting for over 12 weeks. On January 1, 2013, only 3% of patients (6,547 of 209,080) were waiting for over 12 weeks. This grew to 27% on December 31, 2019 (75,322 of 281,235 totals). During the pandemic, the percentage of patients who waited for longer than 12 weeks increased substantially, reaching 60% on December 31, 2023 (317,070 of 529,321 totals).

Supplementary Materials

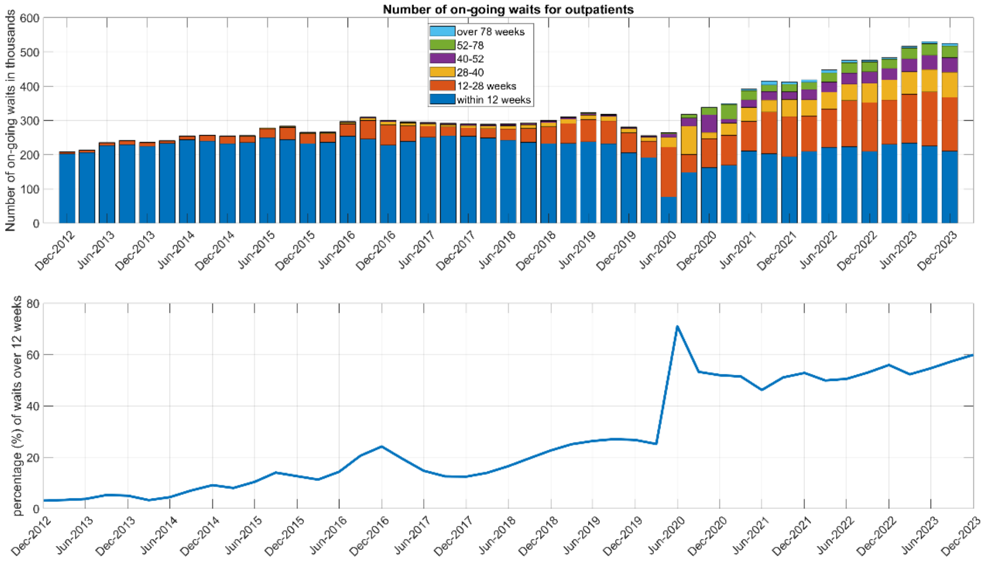

Figure S14: Distribution of ongoing outpatient cases in terms of how long the patients have been waiting for (upper panel) and the percentage of cases that have been waiting for over 12 weeks (lower panel)

8: Distribution of pending cases stratified by NHS Health Board (Inpatients)

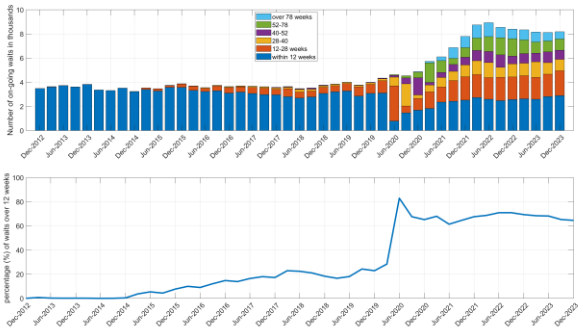

(a) Ayrshire and Arran

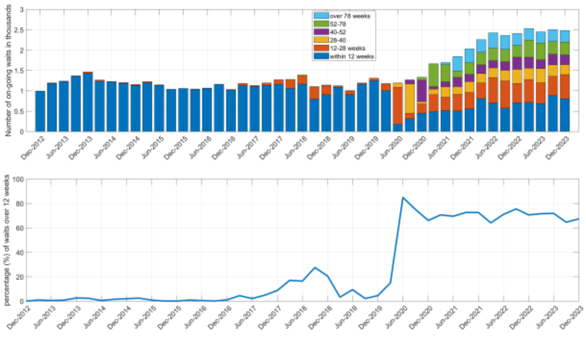

(b) Borders

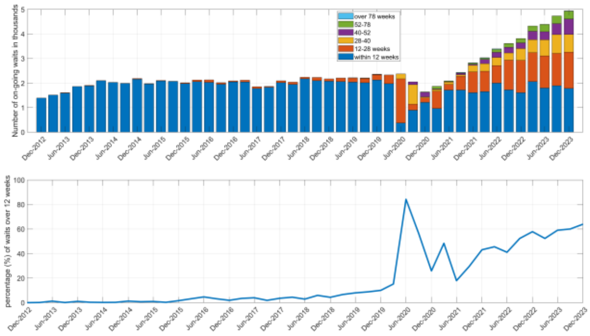

(c) Dumfries and Galloway

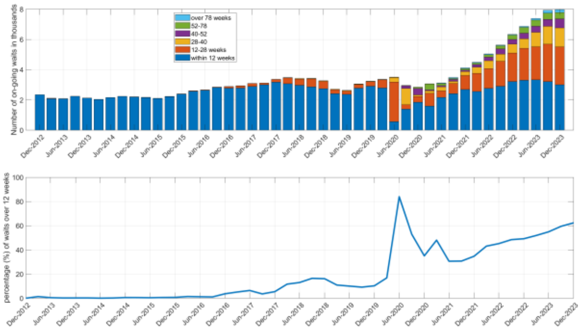

(d) Fife

Supplementary Materials

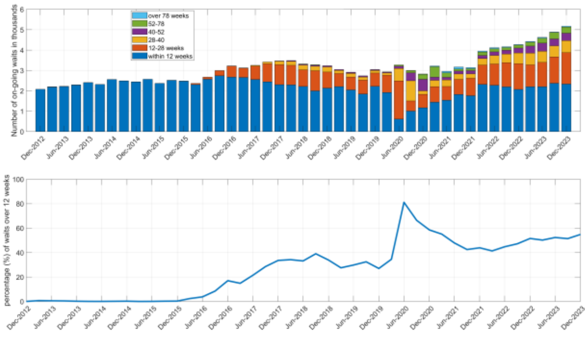

(e) Forth Valley

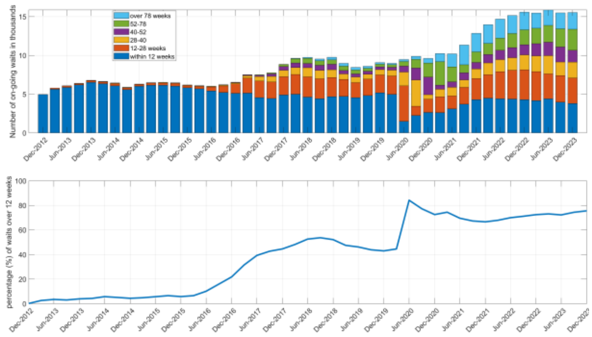

(g) Grampian

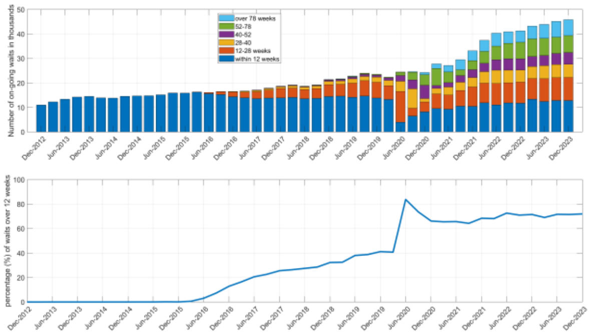

(h) Greater Glasgow and Clyde

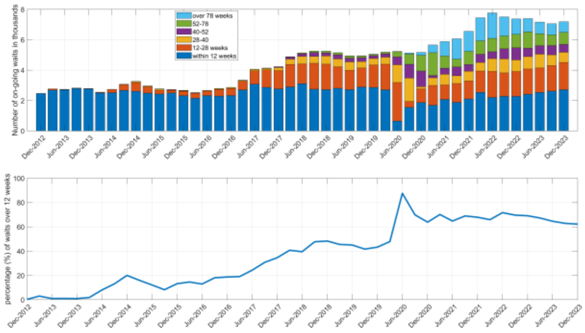

(i) Highland

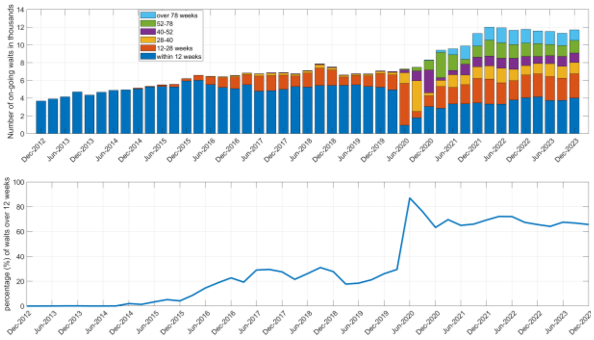

(j) Lanarkshire

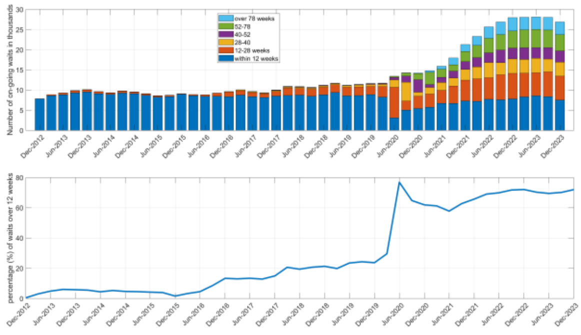

(k) Lothian

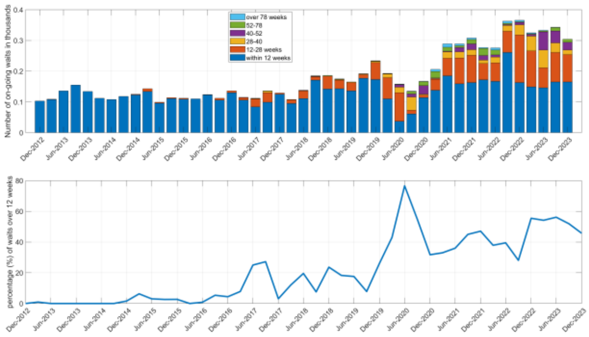

(l) Orkney

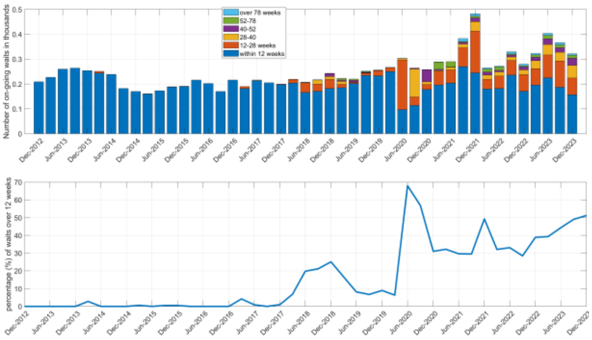

(m) Shetland

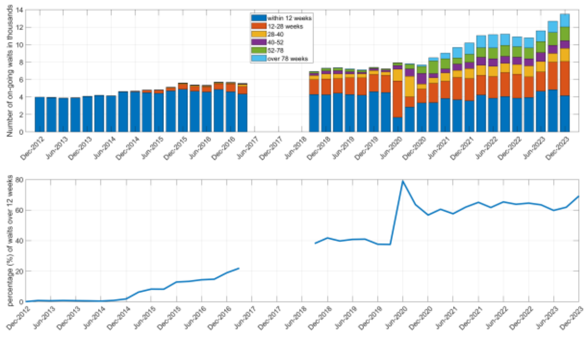

(n) Tayside

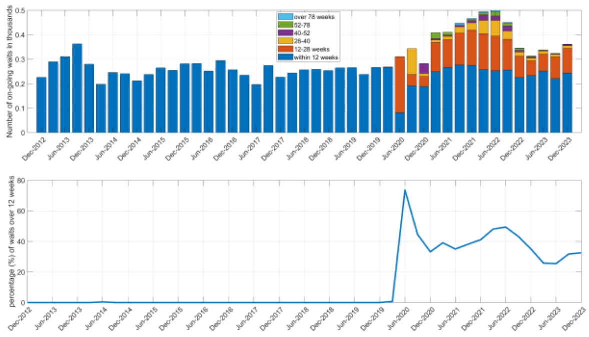

(o) Western Isles

Supplementary Materials

Figure S15: Distribution of pending inpatient/day cases in different regional boards in terms of how long the patients have been waiting for (upper panel) and the percentage of cases that have been waiting for over 12 weeks (lower panel)

Table S7: Total number of pending inpatients/day cases in 2013, 2019, 2023 and the percentage change in numbers comparing 2019 with 2013, and 2023 with 2019.

	**Number of ongoing waits**			**Percentage change (%)**	
**NHS Board**	**2013, mean (95% CI)**	**2019**	**2023**	**2013-2019**	**2019-2023**
Ayrshire and Arran	3704 (3552 ; 3856)	3908 (3730 ; 4087)	8204 (8052 ; 8356)	5.5	109.9
Borders	1317 (1121 ; 1513)	1158 (953 ; 1363)	2494 (2439 ; 2548)	-12.1	115.3
Dumfries and Galloway	1723 (1419 ; 2026)	2249 (2128 ; 2369)	4600 (4137 ; 5062)	30.6	104.5
Forth Valley	2284 (2135 ; 2432)	2938 (2703 ; 3173)	4779 (4262 ; 5297)	28.6	62.7
Grampian	6261 (5576 ; 6946)	8775 (8302 ; 9247)	15583 (15294 ; 15871)	40.1	77.6
Highland	2780 (2723 ; 2837)	5024 (4868 ; 5179)	7204 (6978 ; 7431)	80.7	43.4
Lothian	9552 (8654 ; 10450)	11495 (11125 ; 11866)	27794 (26849 ; 28738)	20.3	141.8
Orkney	134 (103 ; 164)	192 (143 ; 240)	326 (300 ; 353)	43.4	70.4
Shetland	251 (225 ; 278)	238 (207 ; 268)	354 (291 ; 417)	-5.5	49.1
Western Isles	311 (252 ; 370)	259 (237 ; 281)	335 (301 ; 368)	-16.7	29.2
Fife	2144 (2034 ; 2255)	2911 (2452 ; 3371)	7542 (6709 ; 8375)	35.8	159.1
Tayside	3967 (3827 ; 4107)	7280 (7082 ; 7478)	12142 (10241 ; 14042)	83.5	66.8
Greater Glasgow and Clyde	13610 (11994 ; 15225)	23022 (21420 ; 24623)	44557 (42689 ; 46425)	69.2	93.5
Lanarkshire	4276 (3744 ; 4808)	6796 (6482 ; 7109)	11530 (11280 ; 11780)	58.9	69.7

Table S8: Percentage of pending inpatients/day cases in 2013, 2019, 2023 that are over 12 weeks

**NHS Board**	**Percentage of ongoing waits over 12 weeks (95% CI)**		
	**2013**	**2019**	**2023**
NHS Ayrshire and Arran	0 (0 ; 1)	21 (15 ; 26)	67 (64 ; 70)
NHS Borders	1 (0 ; 3)	5 (0 ; 10)	69 (63 ; 75)
NHS Dumfries and Galloway	1 (0 ; 2)	8 (6 ; 11)	59 (51 ; 67)
NHS Forth Valley	0 (0 ; 1)	29 (25 ; 33)	52 (49 ; 55)
NHS Grampian	3 (2 ; 4)	45 (42 ; 48)	74 (71 ; 76)
NHS Highland	2 (0 ; 3)	44 (41 ; 47)	64 (61 ; 68)
NHS Lothian	5 (3 ; 7)	23 (20 ; 26)	71 (69 ; 72)
NHS Orkney	0 (-1 ; 1)	17 (6 ; 29)	52 (45 ; 59)
NHS Shetland	0 (0 ; 0)	10 (3 ; 17)	46 (38 ; 54)
NHS Western Isles	0 (0 ; 0)	0 (0 ; 0)	29 (23 ; 35)
NHS Fife	1 (0 ; 1)	10 (9 ; 11)	57 (50 ; 65)
NHS Tayside	1 (1 ; 1)	40 (37 ; 42)	64 (57 ; 70)
NHS Greater Glasgow and Clyde	0 (0 ; 0)	38 (32 ; 43)	71 (69 ; 73)
NHS Lanarkshire	0 (0 ; 0)	21 (15 ; 27)	66 (64 ; 68)

9: Distribution of pending cases stratified by Specialty (Inpatients)

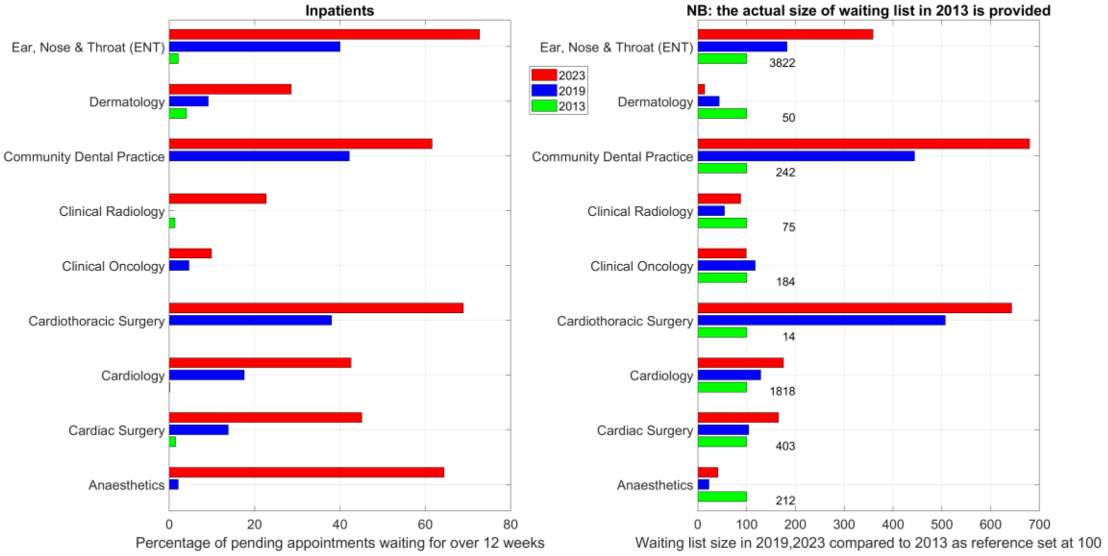

Figure S16: Comparison of ongoing inpatient cases at the end of 2013, 2019 and 2023 by: (left panel) percentage of pending appointments waiting for over 12 weeks; (right panel) relative size of the waiting list using the total size in 2013 as reference. The total size in 2013 is provided in the figure. The specialties covered are: Anaesthetics, Cardiac Surgery, Cardiology, Cardiothoracic Surgery, Clinical Oncology, Clinical Radiology, Community Dental Practice, Dermatology, and Ear, Nose & Throat (ENT).

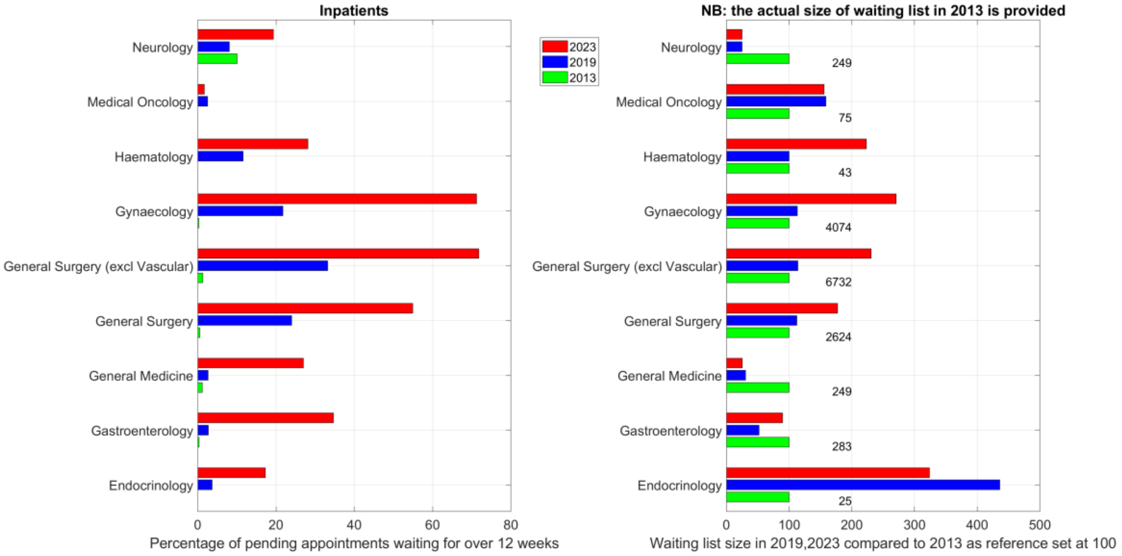

Figure S17: Comparison of ongoing inpatient cases at the end of 2013, 2019 and 2023 by: (left panel) percentage of pending appointments waiting for over 12 weeks; (right panel) relative size of the waiting list using the total size in 2013 as reference. The total size in 2013 is provided in the figure. The specialties covered are: Endocrinology, Gastroenterology, General Medicine, General Surgery, General Surgery (excluding Vascular), Gynaecology,

Supplementary Materials

Haematology, Medical Oncology, and Neurology

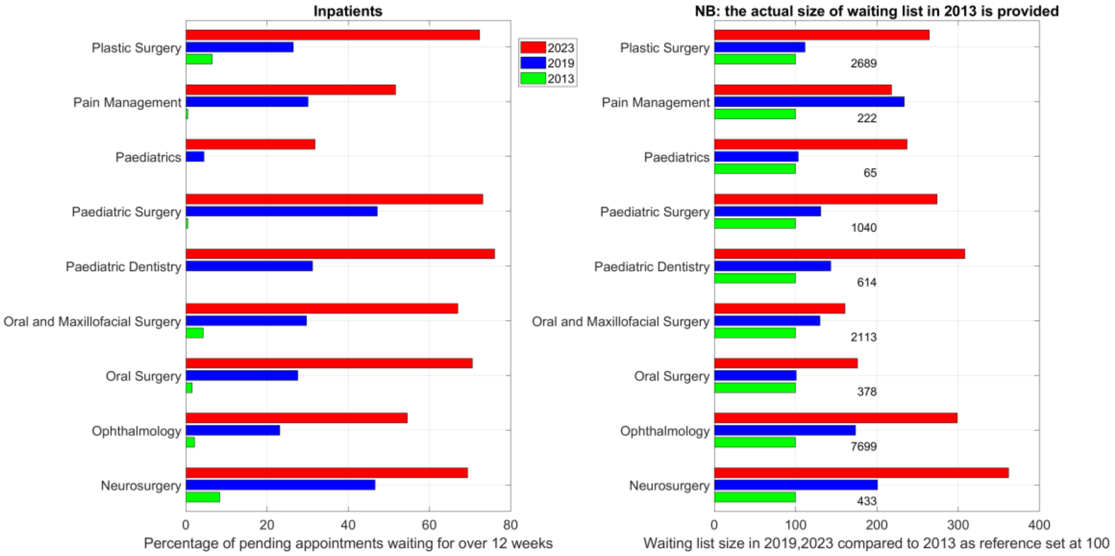

Figure S18: Comparison of ongoing inpatient cases at the end of 2013, 2019 and 2023 by: (left panel) percentage of pending appointments waiting for over 12 weeks; (right panel) relative size of the waiting list using the total size in 2013 as reference. The total size in 2013 is provided in the figure. The specialties covered are: Neurosurgery, Ophthalmology, Oral Surgery, Oral Maxillofacial Surgery, Paediatric Dentistry, Paediatric Surgery, Paediatrics, Pain Management, and Plastic Surgery.

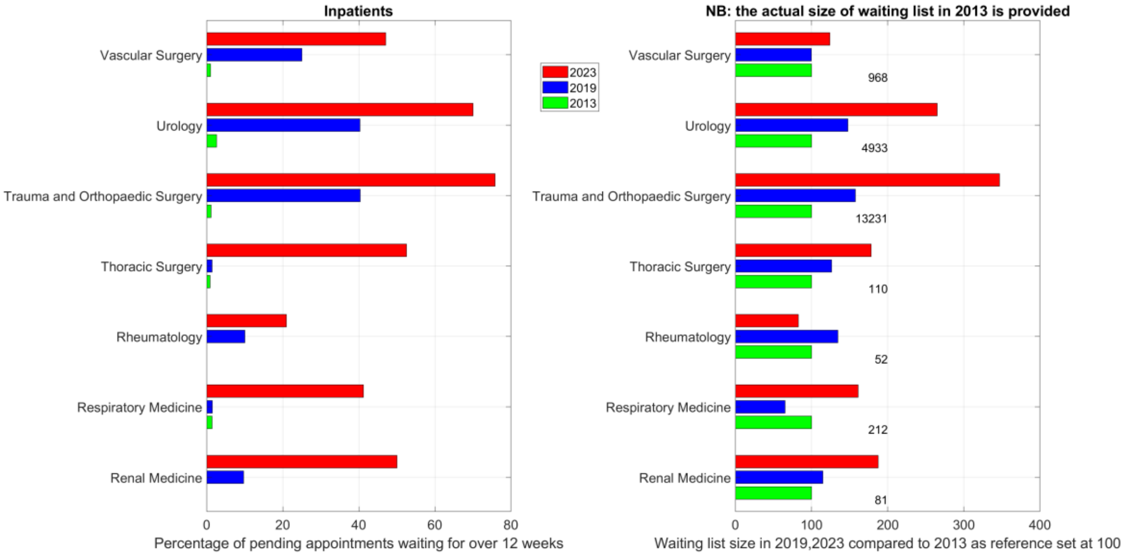

Figure S19: Comparison of ongoing inpatient cases at the end of 2013, 2019 and 2023 by: (left panel) percentage of pending appointments waiting for over 12 weeks; (right panel) relative size of the waiting list using the total size in 2013 as reference. The total size in 2013 is provided in the figure. The specialties covered are: Renal Medicine, Respiratory Medicine, Rheumatology, Thoracic Surgery, Trauma and Orthopaedic Surgery, Urology, and Vascular Surgery.

Supplementary Materials

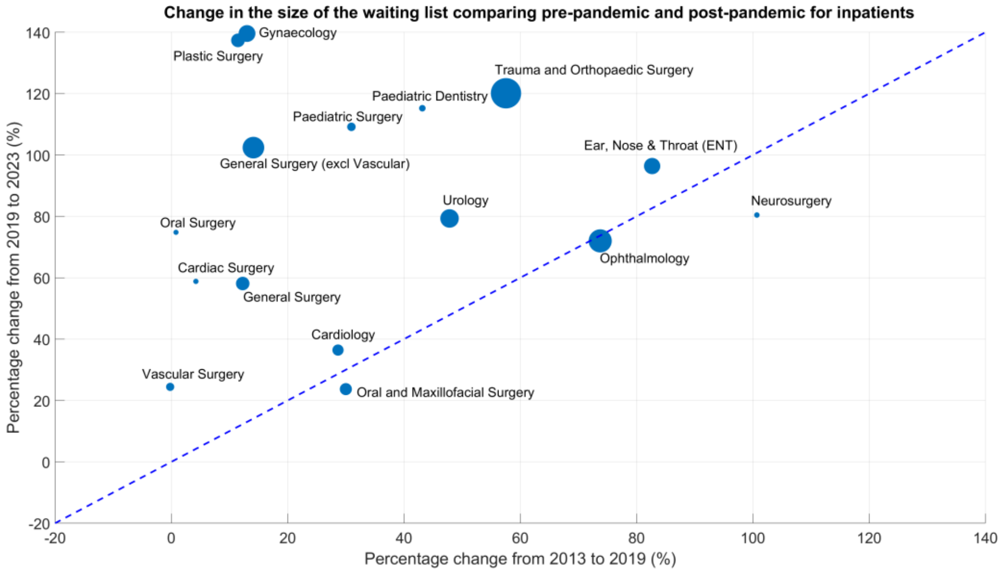

Figure S20: Comparison of the percentage change in the size of the waiting list during the pre-pandemic period (percentage change in the waiting size from the end of 2019 to the end of 2023), and during the pandemic period (percentage change in the waiting size from the end of 2019 to the end of 2023). Any specialty below the dotted line means that the percentage change was greater in the pre-pandemic period compared to the pandemic period, and any points above the dotted line means that there was greater change during the pandemic period. There were 16 specialties with at least 250 pending referrals at the end of 2013 and are included in this plot. The size of the data points is proportional to the size of the waiting list at the end of 2013.

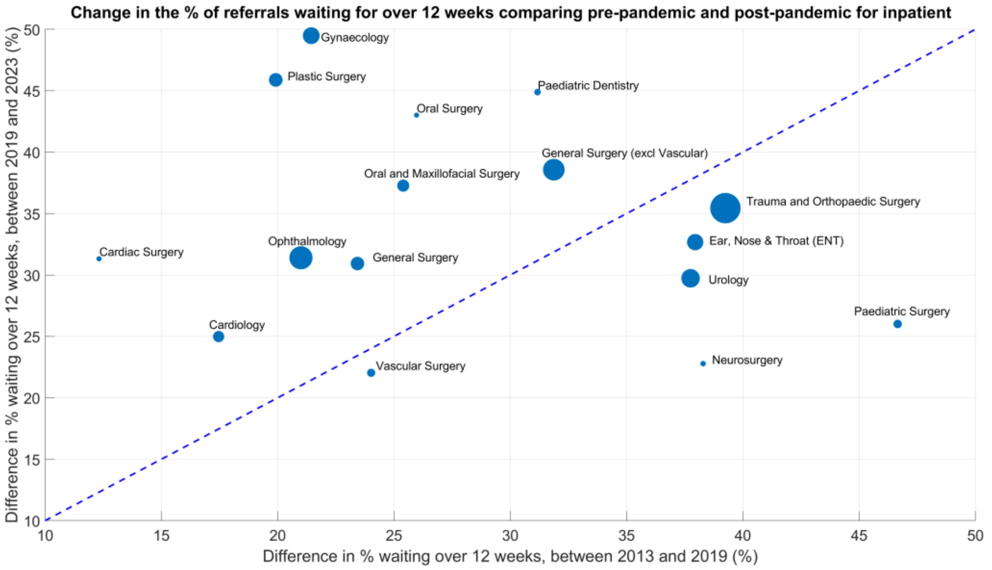

Figure S21: Comparison of the difference in the percentage of referrals waiting for over 12 weeks during the pre-pandemic period (from the end of 2013 to the end of 2019) and the pandemic period (from the end of 2019 to the end of 2023). Any specialty below the dotted line means that the percentage change was greater in the pre-pandemic period compared to the pandemic period, and any points above the dotted line means that there was greater change during the pandemic period. There were 16 specialties with at least 250 pending referrals at the end of 2013 and are included in this plot. The size of the data points is proportional to the size of the waiting list at the end of 2013.

10: Distribution of pending cases stratified by NHS Health Board (Outpatients)

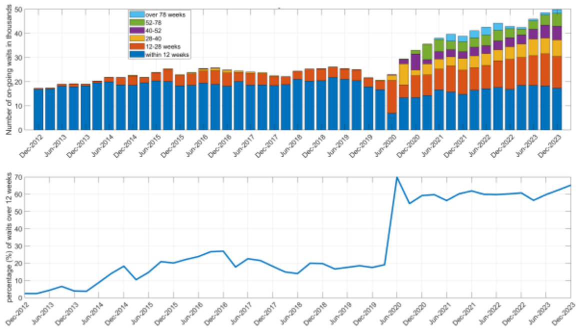

(a) Ayrshire and Arran

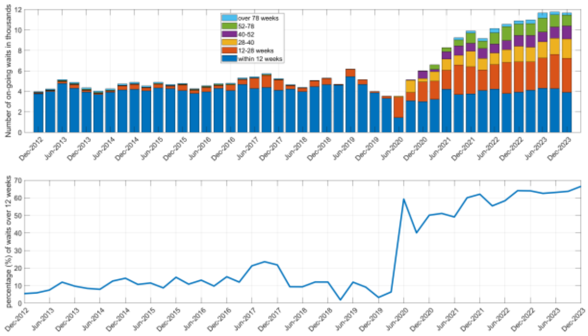

(b) Borders

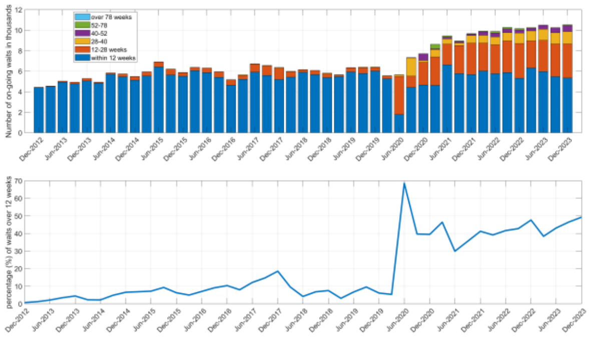

(c) Dumfries and Galloway

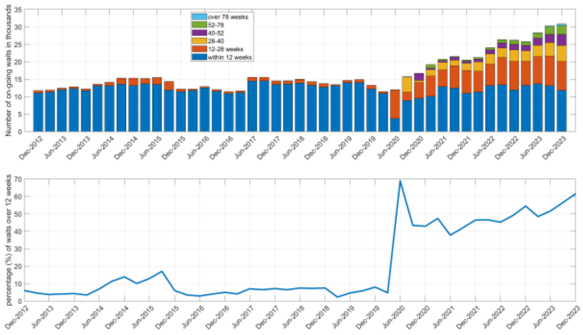

(d) Fife

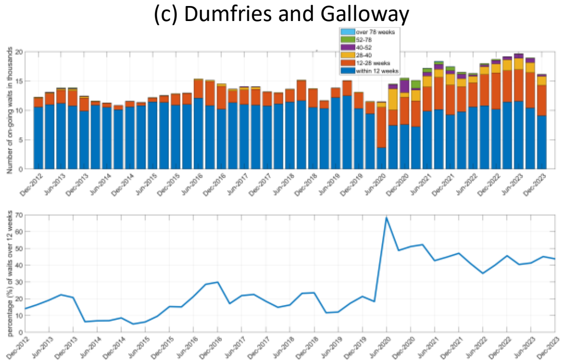

(e) Forth Valley

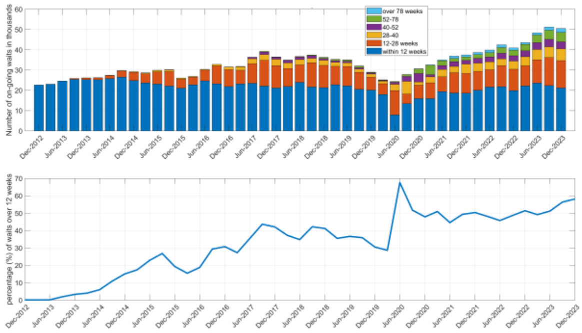

(g) Grampian

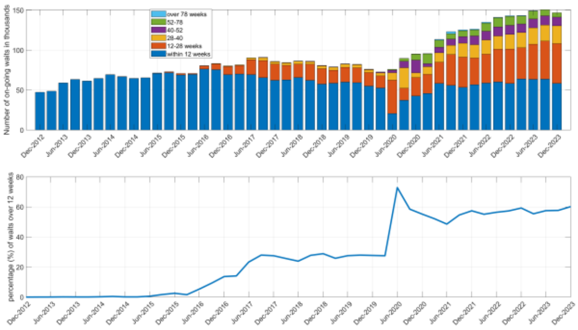

(h) Greater Glasgow and Clyde

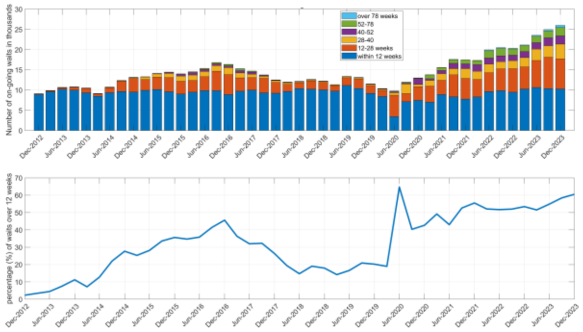

(i) Highland

Supplementary Materials

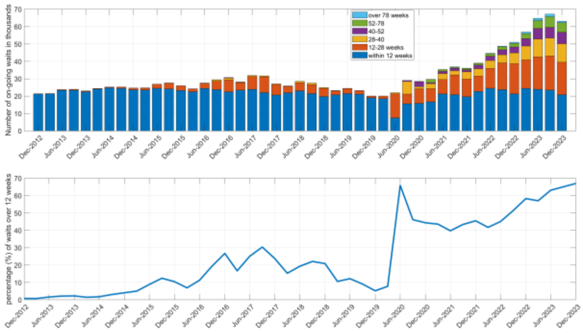

(j) Lanarkshire

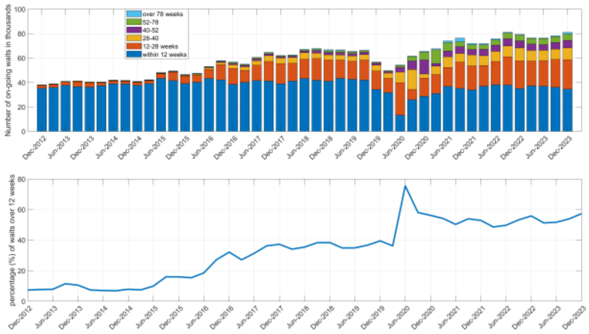

(k) Lothian

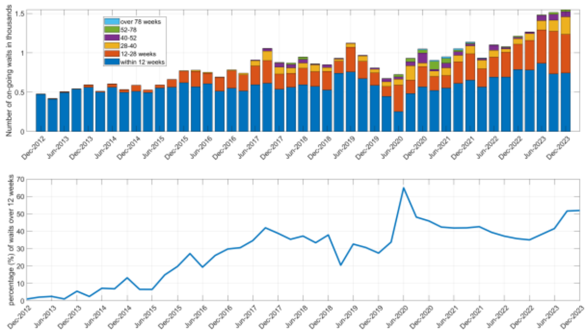

(l) Orkney

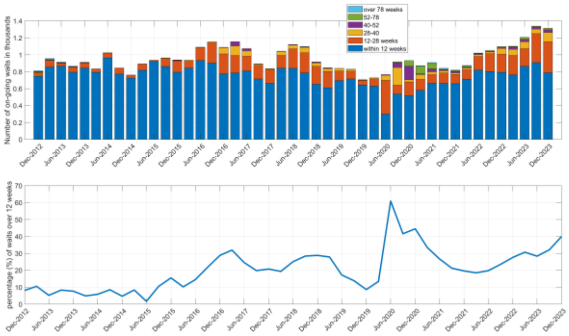

(m) Shetland

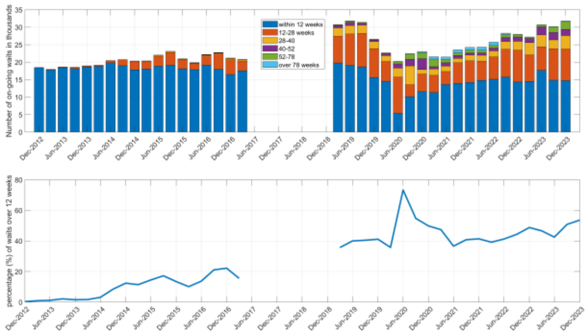

(n) Tayside

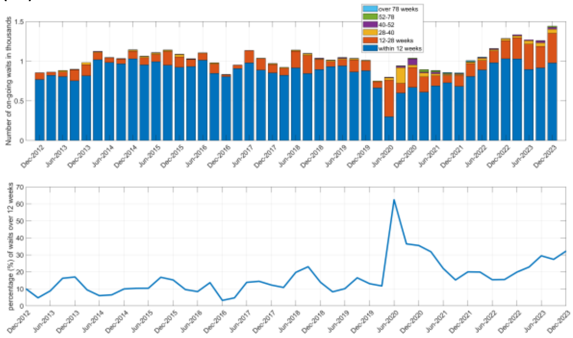

(o) Western Isles

Figure S22: Distribution of pending outpatient cases in different regional boards in terms of how long the patients have been waiting for (upper panel) and the percentage of cases that have been waiting for over 12 weeks (lower panel)

Table S9: Total number of pending outpatient cases in 2013, 2019, 2023 and the percentage change in numbers comparing 2019 with 2013, and 2023 with 2019.

	**Number of ongoing waits, mean (95% CI)**			**Percentage change, %**	
**NHS Board**	**2013**	**2019**	**2023**	**2013-2019**	**2019-2023**
Ayrshire and Arran	18666 (17377 ; 19954)	24589 (21423 ; 27755)	46739 (41543 ; 51935)	31.7	90.1
Borders	4663 (3968 ; 5359)	5010 (3550 ; 6469)	11525 (10948 ; 12102)	7.4	130.1
Dumfries and Galloway	4960 (4479 ; 5441)	6208 (5620 ; 6795)	10387 (10138 ; 10636)	25.2	67.3
Fife	12344 (11710 ; 12977)	14072 (12745 ; 15398)	28824 (25165 ; 32483)	14.0	104.8
Forth Valley	13318 (12259 ; 14378)	13433 (11166 ; 15699)	18486 (15962 ; 21009)	0.9	37.6
Grampian	24916 (22682 ; 27150)	32812 (28084 ; 37540)	48417 (42943 ; 53891)	31.7	47.6
Greater Glasgow and Clyde	58106 (47660 ; 68551)	80126 (75176 ; 85076)	147582 (142508 ; 152657)	37.9	84.2
Highland	10474 (9846 ; 11101)	12346 (10679 ; 14013)	23915 (20566 ; 27264)	17.9	93.7

Lanarkshire	23037 (21233 ; 24841)	22745 (19757 ; 25733)	63030 (56019 ; 70040)	-1.3	177.1
Lothian	40437 (38759 ; 42115)	63817 (56232 ; 71403)	78105 (74664 ; 81546)	57.8	22.4
Orkney	517 (400 ; 633)	959 (752 ; 1166)	1453 (1251 ; 1655)	85.6	51.5
Shetland	912 (853 ; 971)	806 (698 ; 913)	1240 (1067 ; 1413)	-11.7	53.9
Tayside	18535 (17871 ; 19198)	30136 (26288 ; 33983)	29986 (26865 ; 33106)	62.6	-0.5
Western Isles	907 (824 ; 991)	1028 (1001 ; 1054)	1327 (1194 ; 1460)	13.3	29.1

Table S10: Percentage of pending outpatient cases in 2013, 2019, 2023 that are over 12 weeks

**NHS Board**	**Percentage of ongoing waits over 12 weeks, % (95% CI)**		
	**2013**	**2019**	**2023**
NHS Ayrshire and Arran	4 (2 ; 7)	18 (16 ; 19)	61 (55 ; 67)
NHS Borders	9 (5 ; 13)	7 (-1 ; 14)	64 (61 ; 67)
NHS Dumfries and Galloway	3 (1 ; 5)	6 (2 ; 11)	44 (37 ; 52)
NHS Forth Valley	20 (16 ; 24)	16 (8 ; 23)	43 (39 ; 46)
NHS Grampian	1 (-1 ; 4)	35 (30 ; 39)	54 (47 ; 60)
NHS Highland	7 (1 ; 12)	18 (13 ; 23)	56 (50 ; 63)
NHS Lothian	9 (6 ; 12)	37 (33 ; 40)	54 (49 ; 58)
NHS Orkney	3 (0 ; 6)	28 (19 ; 36)	46 (35 ; 57)
NHS Shetland	8 (4 ; 11)	17 (4 ; 30)	33 (25 ; 41)
NHS Western Isles	12 (2 ; 21)	12 (6 ; 18)	28 (22 ; 34)
NHS Fife	4 (4 ; 5)	5 (1 ; 9)	55 (45 ; 64)
NHS Tayside	1 (1 ; 2)	39 (35 ; 43)	48 (41 ; 56)
NHS Greater Glasgow and Clyde	0 (0 ; 0)	27 (26 ; 29)	58 (55 ; 61)
NHS Lanarkshire	2 (1 ; 3)	9 (5 ; 14)	63 (56 ; 70)

11: Distribution of pending cases stratified by Specialty (Outpatients)

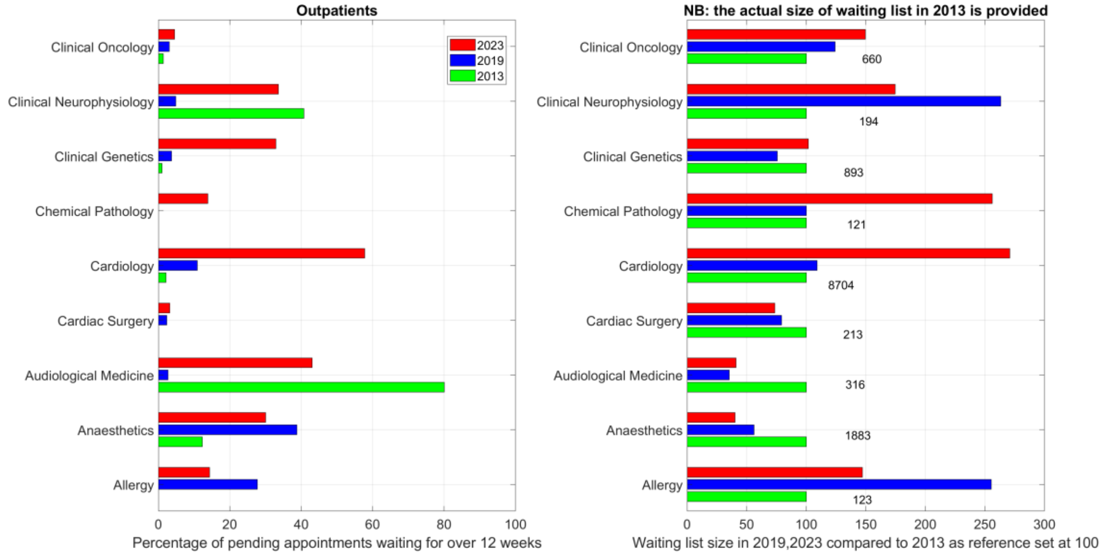

Figure S23: Comparison of ongoing outpatient cases at the end of 2013, 2019 and 2023 by: (left panel) percentage of pending appointments waiting for over 12 weeks; (right panel) relative size of the waiting list using the total size in 2013 as reference. The total size in 2013 is provided in the figure. The specialties covered are: Allergy, Anaesthetic, Audiological Medicine, Cardiac Surgery, Cardiology, Chemical Pathology, Clinical Genetics, Clinical Neurophysiology, and Clinical Oncology.

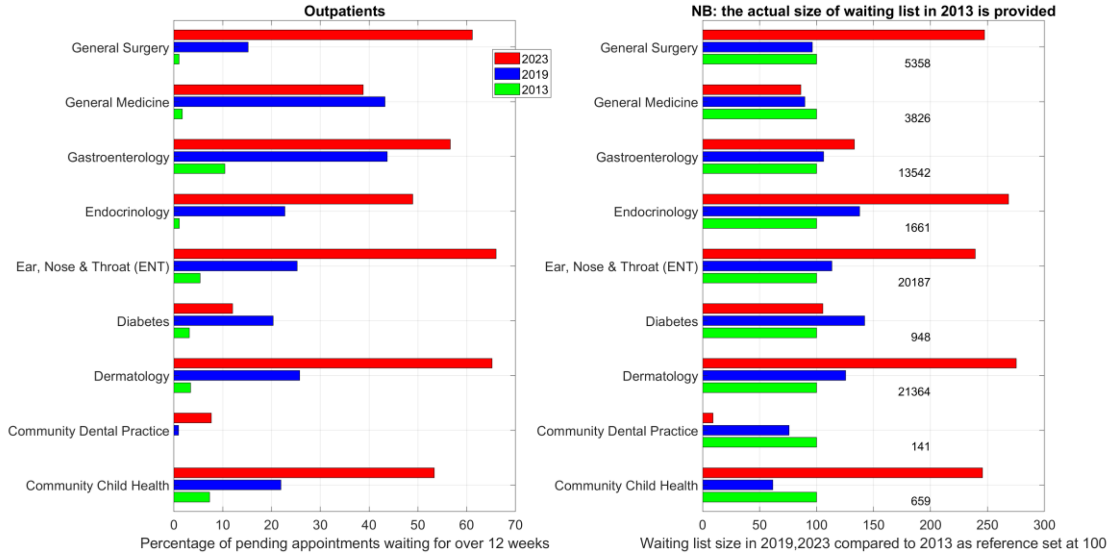

Figure S24: Comparison of ongoing outpatient cases at the end of 2013, 2019 and 2023 by: (left panel) percentage of pending appointments waiting for over 12 weeks; (right panel) relative size of the waiting list using the total size in 2013 as reference. The total size in 2013 is provided in the figure. The specialties covered are: , Community Child Health, Community Dental Practice, Dermatology, Diabetes, Ear, Nose & Throat (ENT), Endocrinology, Gastroenterology, General Medicine, and General Surgery.

Supplementary Materials

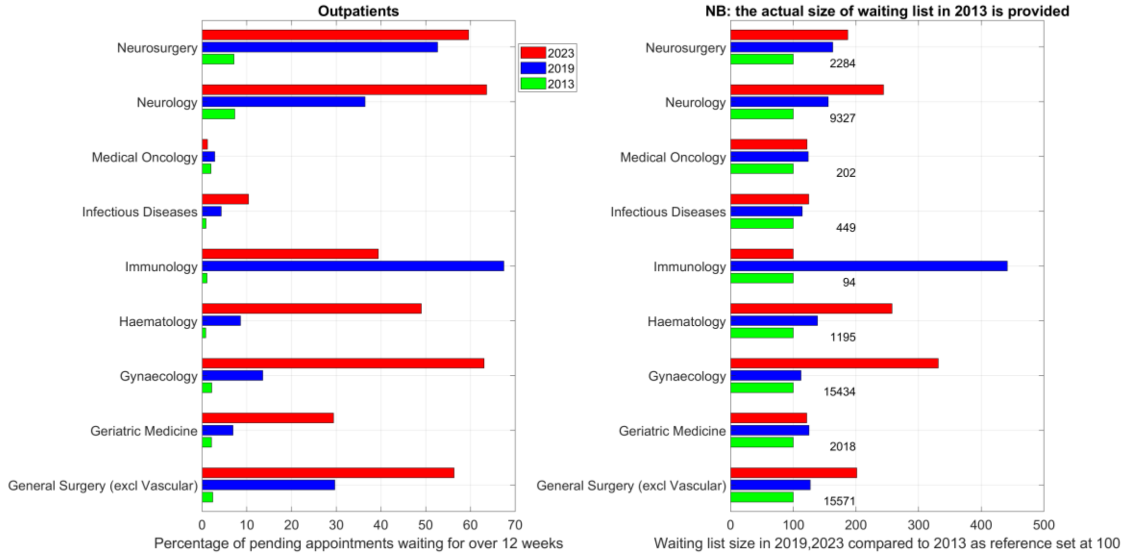

Figure S25: Comparison of ongoing outpatient cases at the end of 2013, 2019 and 2023 by: (left panel) percentage of pending appointments waiting for over 12 weeks; (right panel) relative size of the waiting list using the total size in 2013 as reference. The total size in 2013 is provided in the figure. The specialties covered are: General Surgery (excluding Vascular), Geriatric Medicine, Gynaecology, Haematology, Immunology, Infectious Diseases, Medical Oncology, Neurology, and Neurosurgery.

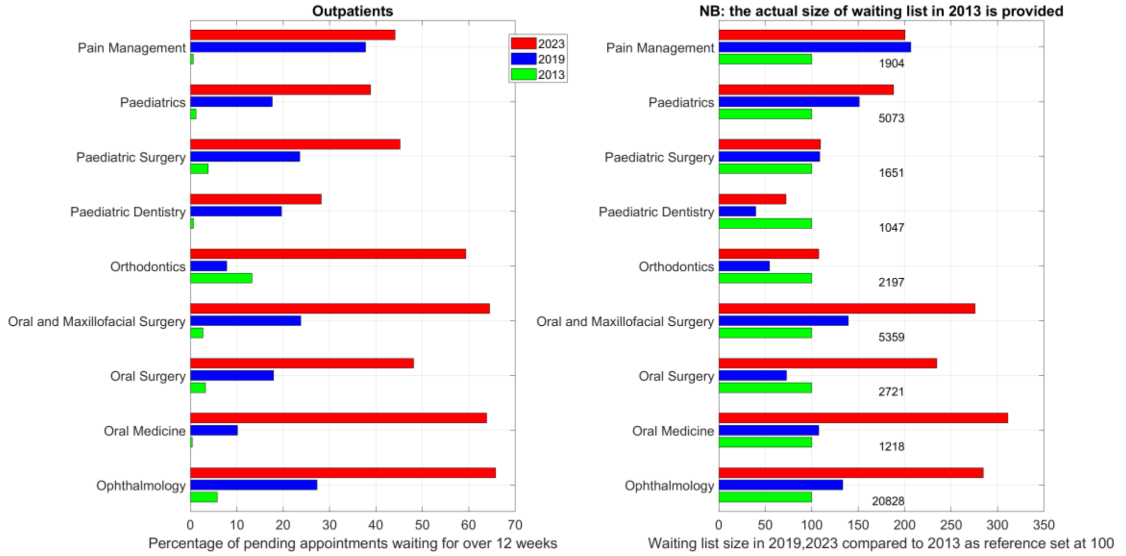

Figure S26: Comparison of ongoing outpatient cases at the end of 2013, 2019 and 2023 by: (left panel) percentage of pending appointments waiting for over 12 weeks; (right panel) relative size of the waiting list using the total size in 2013 as reference. The total size in 2013 is provided in the figure. The specialties covered are: Ophthalmology, Oral Medicine, Oral Surgery, Oral Maxillofacial Surgery, Orthodontics, Paediatric Dentistry, Paediatric Surgery, Paediatrics, and Pain Management.

Supplementary Materials

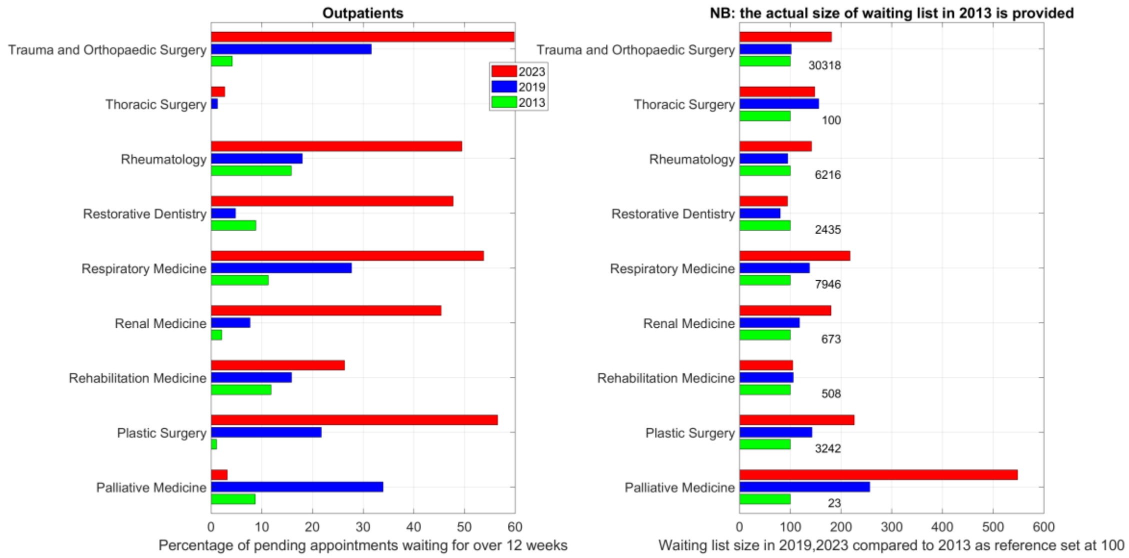

Figure S27: Comparison of ongoing outpatient cases at the end of 2013, 2019 and 2023 by: (left panel) percentage of pending appointments waiting for over 12 weeks; (right panel) relative size of the waiting list using the total size in 2013 as reference. The total size in 2013 is provided in the figure. The specialties covered are: Palliative Medicine, Plastic Surgery, Rehabilitation Medicine, Renal Medicine, Respiratory Medicine, Restorative Dentistry, Rheumatology, Thoracic Surgery, and Trauma and Orthopaedic Surgery.

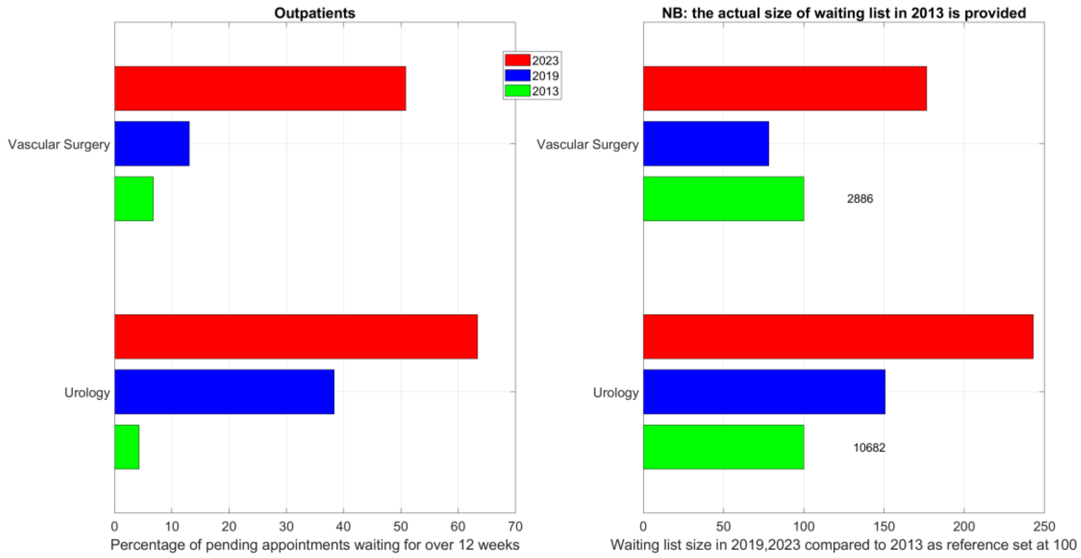

Figure S28: Comparison of ongoing outpatient cases at the end of 2013, 2019 and 2023 by: (left panel) percentage of pending appointments waiting for over 12 weeks; (right panel) relative size of the waiting list using the total size in 2013 as reference. The total size in 2013 is provided in the figure. The specialties covered are: Urology, and Vascular Surgery.

Supplementary Materials

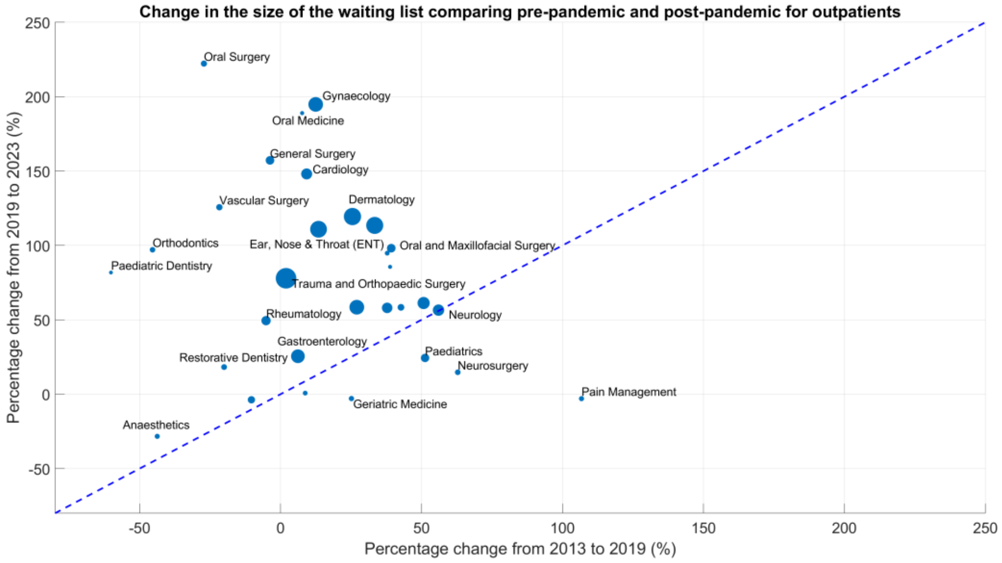

Figure S29: Comparison of the percentage change in the size of the waiting list during the pre-pandemic period (percentage change in the waiting size from the end of 2019 to the end of 2023), and during the pandemic period (percentage change in the waiting size from the end of 2019 to the end of 2023). Any specialty below the dotted line means that the percentage change was greater in the pre-pandemic period compared to the pandemic period, and any points above the dotted line means that there was greater change during the pandemic period. The names of some specialties are not shown to avoid clutter. There were 38 specialties with at least 1,000 pending referrals at the end of 2013 and are included in this plot. The size of the data points is proportional to the size of the waiting list at the end of 2013.

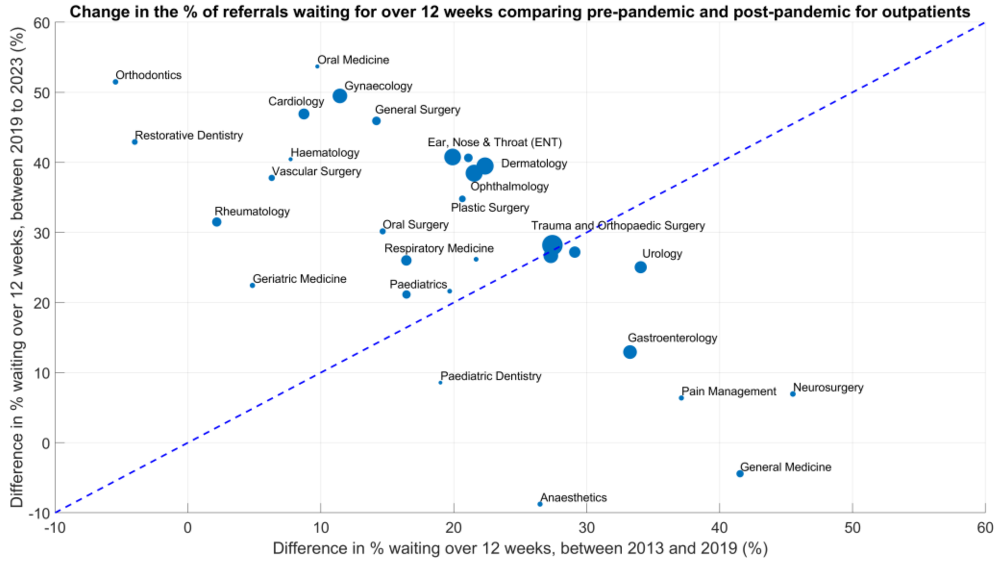

Figure S30: Comparison of the difference in the percentage of referrals waiting for over 12 weeks during the pre-pandemic period (from the end of 2013 to the end of 2019) and the pandemic period (from the end of 2019 to the end of 2023). Any specialty below the dotted line means that the percentage change was greater in the pre-pandemic period compared to the pandemic period, and any points above the dotted line means that there was greater change during the pandemic period. There were 16 specialties with at least 250 pending referrals at the end of 2013 and are included in this plot. The names of some specialties are not shown to avoid clutter. There were 38 specialties with at least 1,000 pending referrals at the end of 2013 and are included in this plot. The size of the data points is proportional to the size of the waiting list at the end of 2013.

12: Assessment of progress against the Government's NHS Recovery Plan

The NHS Recovery Plan published by the Scottish Government in August 2021 (https://www.gov.scot/binaries/content/documents/govscot/publications/strategy-plan/2021/08/nhs-recovery-plan/documents/nhs-recovery-plan-2021-2026/nhs-recovery-plan-2021-2026/govscot%3Adocument/nhs-recovery-plan-2021-2026.pdf) contained the capacity increase plans for inpatients (Table S11) and outpatients (Table S12) reproduced under the Open Government Licence v3.0. (https://www.nationalarchives.gov.uk/doc/open-government-licence/version/3).

Table S11: Reproduced from the NHS Recovery Plan published by the Scottish Government, and shared under the UK Open Government licence, showing the planned increase in admitted cases (referred to as inpatients in our study for brevity) over four years.

Additional Inpatient and Day case Activity	2022/23	2023/24	2024/25	2025/26
National Treatment Centres	12,000	19,000	25,000	40,000
Additional activity at NHS board level	15,500	15,500	15,500	15,500
TOTAL	27,500	34,500	40,500	55,500

Table S12: Reproduced from the NHS Recovery Plan published by the Scottish Government, and shared under the UK Open Government licence, showing the planned increase in outpatient cases (referred to as inpatients in our study for brevity) over four years

Outpatient activity	2022/23	2023/24	2024/25	2025/26
Increases in hospital outpatient capacity	8,000	12,000	16,000	20,000
Releasing activity through redesign of care pathways	50,000	70,000	90,000	120,000
TOTAL	58,000	82,000	106,000	140,000

Inpatients

For the period 2022-2023 (corresponds to April 2022 – March 2023), the plan envisioned an increase of 27,500 inpatient cases. Assuming uniform increase across the four quarters, this amounts to (27,500/4) 6,875 cases per quarter. According to the plan therefore, the inpatient capacity must rise to (67,500+6,875) 74,375 per quarter. For the period 2023-2024 (corresponds to April 2023 – March 2024), the planned increase was 34,500. This amounts to 8,625 cases per quarter. According to the plan therefore, the inpatient capacity must rise to (67,500+8,625) 76,125 per quarter. Overall, the mean planned increase per quarter over the 7 quarters (April 2022 – December 2023) is ((6,875*4 + 8,625*3)/7) 7,625 cases. The actual per-quarter increase during these 7 quarters was 1,811. We estimated the actual per-quarter increase by fitting a straight line and taking the gradient to be the mean capacity increase per quarter (see Figure S31). Table S13 provides a summary of these estimations and provides a quarter-by-quarter comparison of the total planned capacity and the actual capacity observed in the 7 quarters (April 2022 – December 2023).

Supplementary Materials

Table S13: Quarter-by-quarter comparison of the planned capacity and the actual capacity observed during 7 quarters (April 2022 – December 2023) for inpatients across Scotland. Note that the actual capacity for the purposes of this comparison refers to the total cases treated only.

**Baseline capacity (pre-pandemic):** 270,000/year; 67,500/quarter			
**Period**	**Capacity Increase Target**	**Total Planned Capacity**	**Actual Capacity Observed**
April – June 2022	6,875	74,375	49,862
July – September 2022	6,875	74,375	54,008
October – December 2022	6,875	74,375	56,353
January – March 2023	6,875	74,375	57,688
April – June 2023	8,625	76,125	59,241
July – September 2023	8,625	76,125	59,488
October – December 2023	8,625	76,125	62,152
**Mean planned per quarter increase in 7 quarters (April 2022 – December 2023):** 7,625<br>**Mean observed per quarter increase in 7 quarters (April 2022 – December 2023):** 1,811<br>**Shortfall in per-quarter increase over the 7 quarters:** 5,814 (76%)			

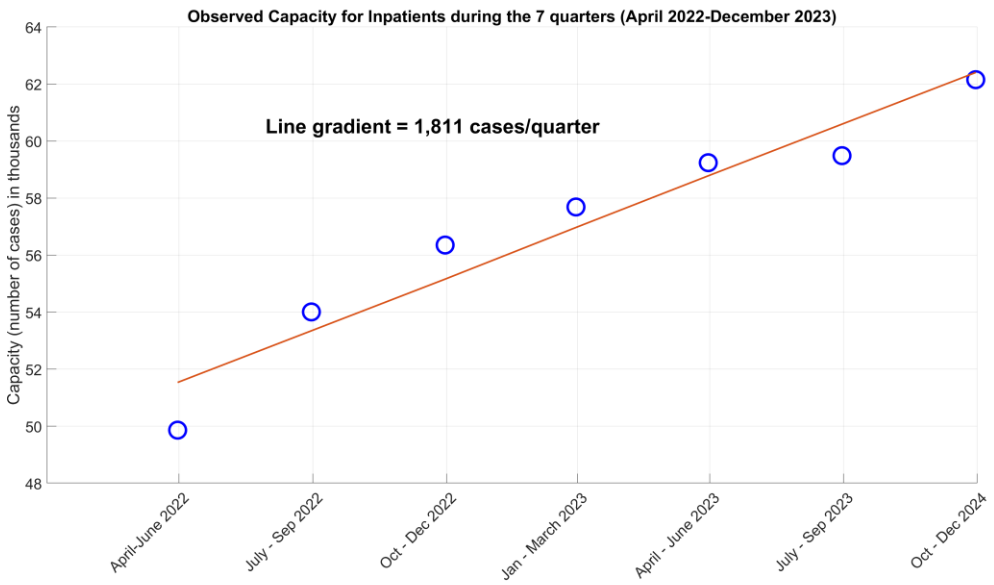

Figure 31: Observed capacity and best-fit line to estimate the per-quarter increase in capacity over the 7 quarters for inpatients

Outpatients

For the period 2022-2023 (corresponds to April 2022 – March 2023), the plan envisioned an increase of 58,000 outpatient cases. Assuming uniform increase across the four quarters, this amounts to (58,000/4) 14,500 cases per quarter. According to the plan therefore, the outpatient capacity must rise to (350,000+14,500) 364,500 per quarter. For the period 2023-2024 (corresponds to April 2023 – March 2024), the planned increase was 82,000. This amounts to 20,500 cases per quarter. According to the plan therefore, the outpatient capacity must rise to (350,000+20,500) 370,500 per quarter. Overall, the mean planned increase per quarter over the 7 quarters (April 2022 – December 2023) was ((14,500*4 + 20,500*3)/7) 17,071 cases. The actual per-quarter increase during these 7 quarters was 2,193 (see Figure S32, where the gradient of the best-fit line was taken to be the mean per-quarter increase in capacity during the 7 quarters). Table S14 provides a summary of these estimations and provides a quarter-by-

quarter comparison of the total planned capacity and the actual capacity observed in the 7 quarters (April 2022 – December 2023).

Table S14: Quarter-by-quarter comparison of the planned capacity and the actual capacity observed during 7 quarters (April 2022 – December 2023) for outpatients across Scotland. Note that the actual capacity for the purposes of this comparison refers to the total cases treated only.

**Baseline capacity (pre-pandemic): 1.4 million/year; 350,000/quarter**			
**Period**	**Capacity Increase Target**	**Total Planned Capacity**	**Actual Capacity Observed**
April – June 2022	14,500	364,500	298,568
July – September 2022	14,500	364,500	301,666
October – December 2022	14,500	364,500	309,695
January – March 2023	14,500	364,500	316,589
April – June 2023	20,500	370,500	303,184
July – September 2023	20,500	370,500	310,954
October – December 2023	20,500	370,500	315,011
**Mean planned per quarter increase in 7 quarters (April 2022 – December 2023):** 17,071<br>**Mean observed per quarter increase in 7 quarters (April 2022 – December 2023):** 2,193<br>**Shortfall in per-quarter increase over the 7 quarters:** 14,878 (87%)			

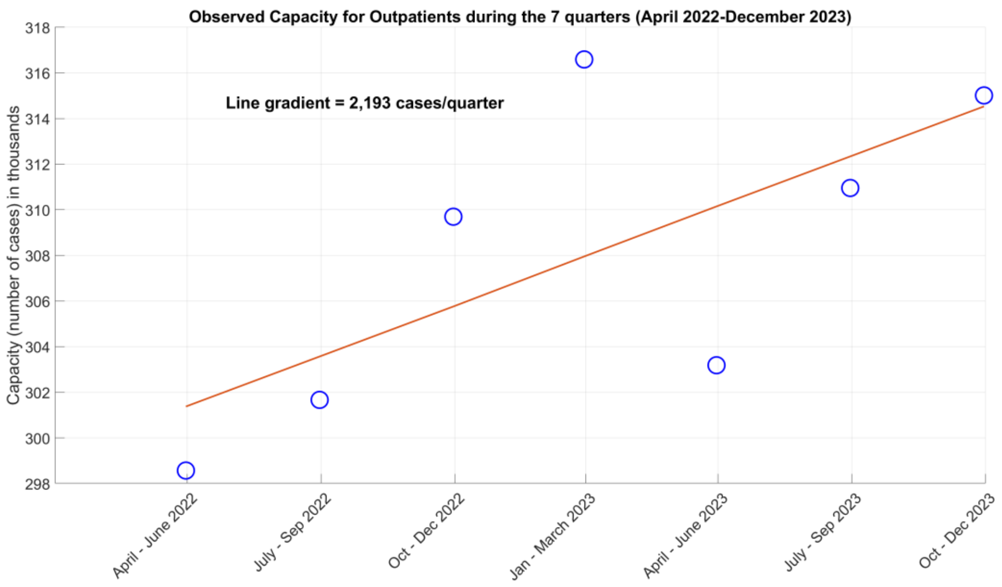

Figure S32: Observed capacity and best-fit line to estimate the per-quarter increase in capacity over the 7 quarters for inpatients

13: Description of the code repository

The code repository is publicly available on GitHub: https://github.com/syedahmar/ElectiveCare-Scotland. While the detailed ReadMe file describing the code and data will be made available upon publication, we have included a brief repository description here for reviewers who may wish to inspect the code and data in advance.

Supplementary Materials

Data

All the data files (except the Matlab format data) is available in the 'data' folder. All the original raw data is made available under the under the UK Open Government Licence (https://www.nationalarchives.gov.uk/doc/open-government-licence/version/3/). The original raw data downloaded from the Public Health Scotland's "Stage of Treatment Waiting Times" are:

CompletedWaits_May2024.csv: data on capacity (i.e. cases that are dealt with)
OngoingWaits_May2024.csv: data on pending referrals (i.e. cases that are waiting for treatment)
AdditionsRemovals_May2024.csv: data on additions (i.e. demand) and removals (i.e. capacity)
Distribution_OngoingWaits_May2024.csv: time distribution of pending referrals
Distribution_CompletedWaits_May2024.csv: time distribution of cases dealt with

From these raw data, we derived several datasets using the "**ProcessRawData.Rmd**". This allowed us to easily select/filter datasets when analysing them for various strata. We use the dplyr package, a convenient choice for filtering/selecting. We subsequently import this data into Matlab and save it as a Matlab data file (stored with the extension ".mat").

Code

GetModelParameters_final.m: This file can be used to learn a new VARX model. You can select whether you want the data overall, or whether you want it stratified by elective type (inpatients; outpatients). We have already saved the optimal models learnt. These are 'LearnedModel_AR14_Overall',' 'LearnedModel_AR14_Inpatients', and 'LearnedModel_AR14_Outpatientspatients'.

HealthcareDisruption_Projections_Scotland_final.m: This is the main file that can be used to plot the existing pending referrals, load the learned model, make projections and then plot them. This file calls the **Get_Projections.m** file that has the code for iteratively computing the projections.

Plot_OngoingDistribution.m: This file extracts the time distribution of the pending cases and creates a two-panel plot where the upper panel shows the histogram of time distribution, and the lower panel shows the percentage of cases that have been waiting for over 12 weeks.

Plot_CompletedDistribution.m: This file extracts the time distribution of the completed cases and creates a two-panel plot where the upper panel shows the histogram of time distribution, and the lower panel shows the percentage of cases that were seen within 12 weeks.

Get_SummaryStatistics.m: This file extracts various summary statistics for the years of interest (2013, 2019 and 2023).

GetCI_Adjust_Seasonality.R: This file written in R is used to correctly compute the 95% confidence interval for quarterly means.

Additional files for plotting and a detailed ReadMe will be made available upon publication.

14: Comparison of Scotland with England

For the data from England, we used the referral to treatment (RTT) time data provided by NHS England (https://www.england.nhs.uk/statistics/statistical-work-areas/rtt-waiting-times/rtt-data-2024-25/). We used the latest available RTT time that provides monthly data on the number of referrals pending, and the number of referrals resolved (admitted is taken to be inpatients, and non-admitted is taken to be outpatients). To ensure an even comparison, we converted the monthly capacity data into quarterly data by aggregating. The relevant RTT data file, and the processed data is available in the code repository file named "RTT-Overview-England.xlsx" and shared under the UK Open Government Licence v3.0. (https://www.nationalarchives.gov.uk/doc/open-government-licence/version/3).

Table S15: Comparison of capacity and backlog between England and Scotland. The values are the quarterly average for the given year, and the backlogs are the total waiting at the end of the given year.

	Scotland	England
**Capacity (Outpatients) in thousands**		
2013 (mean, 95% CI)	348.5 (332.0; 365.0)	2,524.8 (2,441.8; 2,607.9)
2019 (mean, 95% CI)	367.0 (350.5; 383.5)	3,375.2 (3,292.1; 3,458.2)
2023 (mean, 95% CI)	311.3 (292.2; 332.6)	3,478.7 (3,369.5; 3,573.0)
% Change from 2013 to 2019	5% increase	34% increase
% Change from 2019 to 2023	15% decrease	3% increase
**Capacity (Inpatients) in thousands**		
2013 (mean, 95% CI)	84.2 (79.2; 89.1)	923.4 (801.6; 931.6)
2019 (mean, 95% CI)	70.6 (65.7; 75.5)	906.4 (878.7; 934.1)
2023 (mean, 95% CI)	59.3 (52.6; 64.7)	858.6 (822.0; 889.9)
% Change from 2013 to 2019	16% decrease	2% decrease
% Change from 2019 to 2023	16% decrease	5% decrease
**Backlog (Referrals pending for over a year)**		
2013	343	520
2019	3,056	1,699
2023	78,282	337,450
% Change from 2013 to 2019	791% increase	227% increase
% Change from 2019 to 2023	2,462% increase	19,762% increase
